# Going Round in Circles: A Cognitive Bias in Geometric Reasoning

**DOI:** 10.1162/opmi_a_00169

**Published:** 2024-11-22

**Authors:** Yacin Hamami, Marie Amalric

**Affiliations:** Department of Philosophy, Université de Liège, Liège, Belgium; Department of Humanities, Social and Political Sciences, ETH Zürich, Zürich, Switzerland; Institut Jean Nicod, Department of Cognitive Studies, ENS, EHESS, PSL University, CNRS, Paris, France; Laboratory for Developmental Studies, Department of Psychology, Harvard University, Cambridge, MA, USA; Center for Brain/Mind Science (CIMeC), Universita degli studi di Trento, Rovereto, Italy

**Keywords:** deductive reasoning, spatial cognition, cognitive biases, mathematical counter-intuitions

## Abstract

Deductive reasoning is essential to most of our scientific and technological achievements and is a crucial component to scientific education. In Western culture, deductive reasoning first emerged as a dedicated mode of thinking in the field of geometry, but the cognitive mechanisms behind this major intellectual achievement remain largely understudied. Here, we report an unexpected cognitive bias in geometric reasoning that challenges existing theories of human deductive reasoning. Over two experiments involving almost 250 participants, we show that educated adults systematically mistook as valid a set of elementary invalid inferences with points and circles in the Euclidean plane. Our results suggest that people got “locked” on unwarranted conclusions because they tended to represent geometric premisses in specific ways and they mainly relied on translating, but not scaling, the circles when searching for possible conclusions. We conducted two further experiments to test these hypotheses and found confirmation for them. Although mathematical reasoning is considered as the hallmark of rational thinking, our findings indicate that it is not exempt from cognitive biases and is subject to fundamental counter-intuitions. Our empirical investigations into the source of this bias provide some insights into the cognitive mechanisms underlying geometric deduction, and thus shed light on the cognitive roots of intuitive mathematical reasoning.

## INTRODUCTION

Deductive reasoning is essential to the production of scientific and mathematical knowledge. In Western culture, deduction first emerged as a mode of scientific and mathematical thinking in the field of geometry (Kline, 1972; Netz, 1999). Plato (1961) and Kant (1787) claimed that all humans are equipped with a cognitive faculty—often called geometric intuition—allowing them to reach general geometric truths through a pure exercise of the deductive mind. Euclid himself relied on his geometric intuition to make numerous deductions essential to the geometric proofs of his foundational treatise (Euclid, 1959; Hartshorne, 2000). The most famous example occurs in the very first proposition of the *Elements* where Euclid drew the following inference (I_1_):^1^


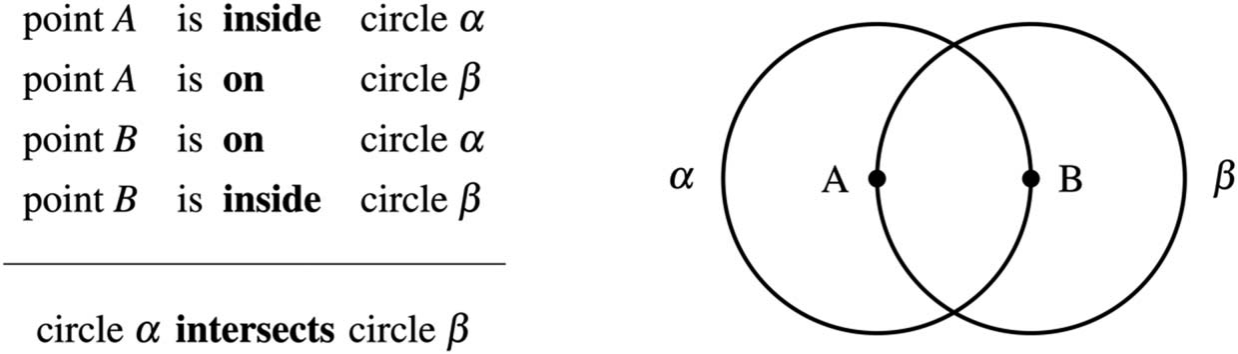


We can all see that if each point is **inside** one of the circles and lies **on** the other one, then the two circles *must* intersect, and this conclusion is as certain for us as it was for the ancient Greeks. Developmental and cross-cultural studies have shown that humans can spontaneously reason about spatial locations (Amalric et al., 2017), shape intruders (Dehaene et al., 2006; Dillon et al., 2013), and metric or spatial relations, for instance in a triangle or between lines in a plane (Izard et al., 2011). But the cognitive mechanisms allowing us to make geometric deductions remain unclear. Here, we report and analyze a cognitive bias in elementary deductive reasoning with points and circles in the Euclidean plane that provides some insights on this issue.

Reasoning deductively means being able to discriminate between valid and invalid inferences. Invalid inferences are those leading to unwarranted conclusions, and so detecting and preventing them is a crucial matter, especially in scientific and mathematical contexts. Here is a typical example of an invalid geometric inference (I_2_):


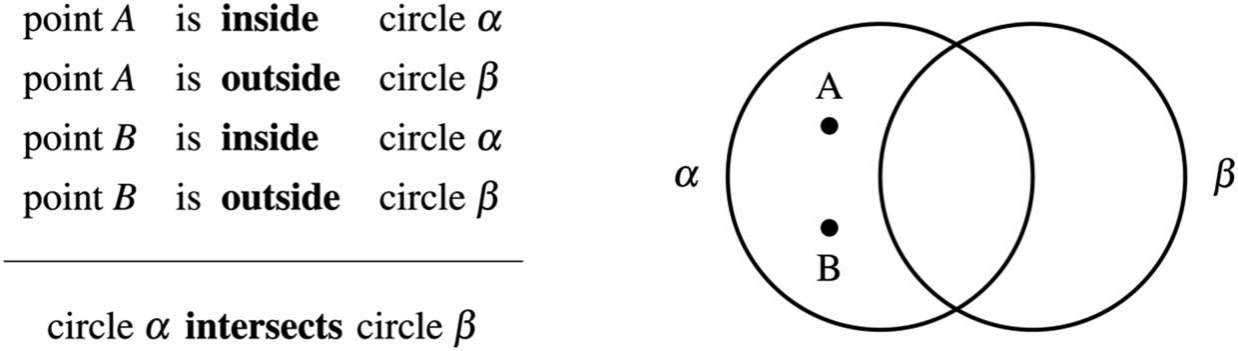


This inference is invalid because the conclusion that “circle *α* intersects circle *β*” is not the only one compatible with the premisses. The premisses can also be met by translating circle *β* (for instance to the right) to produce an alternative configuration where the circles are completely separated. Now consider the following inference (I_3_):


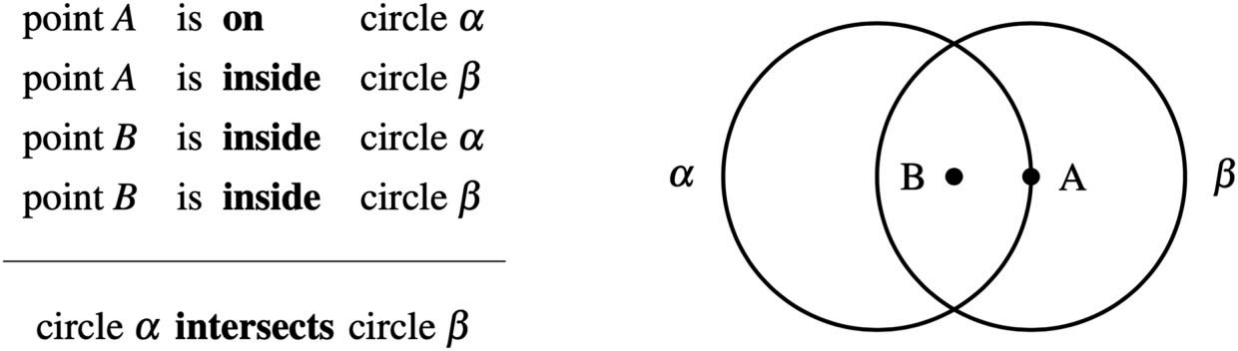


Can you tell whether this inference is valid or invalid? Here, no matter how one would translate the circles, they will always intersect in the above diagram. Does this mean that the conclusion follows from the premisses? If you are like most participants in a previous study (Hamami et al., 2021), you will answer affirmatively and judge this inference as valid. However, if you imagine shrinking circle *α* or enlarging circle *β*, you will realize that circle *α* can be inside circle *β*, and so that the two circles do not have to intersect. This inference is therefore invalid, and yet was almost systematically misjudged as valid by participants who were otherwise able to reliably detect many other invalid geometric inferences. Thus, although mathematical reasoning is often considered as the hallmark of rational thinking, the existence of such systematic errors would indicate that intuitive mathematical reasoning, like many other domains of reasoning (Evans, 1989) and decision-making (Tversky & Kahneman, 1974), may not be exempt from cognitive biases.

The identification of a cognitive bias in deductive mathematical reasoning provides an important opportunity to progress in our understanding of human deductive reasoning. More specifically, it makes it possible to ask whether, and to what extent, contemporary psychological theories of human deduction (Johnson-Laird, 2010; Oaksford & Chater, 2020; Ragni & Knauff, 2013; Rips, 1994) can account for intuitive deductive reasoning with mathematical content—a question all the more relevant given that mathematics is one of the most archetypical contexts in which we reason deductively. These theories make different predictions about the intrinsic difficulty of deductive inferences, and are commonly evaluated in terms of their capacity to predict systematic errors in reasoning. In the mental model theory (Johnson-Laird, 2010), the difficulty of an inference is predicted by the number of mental models compatible with the premisses. This theory would predict that judging validity is more difficult for inference I_3_ than for inference I_2_ since I_2_ admits three mental models compatible with the premisses while inference I_3_ only admits two. The preferred model theory (Ragni & Knauff, 2013) makes an alternative proposal, namely that the difficulty of an inference depends on the distance between the preferred model and the closest countermodel(s). This theory would predict that inferences I_2_ and I_3_ are equally difficult since any alternative to the only model compatible with the premisses is already a countermodel. Rule-based theories of reasoning (Rips, 1994) consider that human reasoning proceeds by the application of mental rules operating on propositions in a mental language akin to a formal language. These theories explain reasoning difficulties by appealing to various factors such as the difficulty of retrieving mental rules from memory, of finding sequences of mental rules allowing to reach a conclusion from a set of premisses, or of judging the absence of such sequences. To make predictions on the difficulty of geometric inferences, rule-based theories of reasoning will require to be further specified by choosing a mental language to encode geometric propositions and a set of mental rules to reason about them. Finally, it seems perfectly possible to develop probabilistic models of geometric reasoning, for instance in the spirit of the new paradigm (Oaksford & Chater, 2020) or in terms of simulation-based statistical models (Hart et al., 2018). The predictions of these theories will, however, depend on how geometric information is represented and operated upon within such frameworks. Thus, existing psychological theories of human deductive reasoning either make different predictions as to the difficulty of elementary geometric inferences such as I_2_ and I_3_, or would require further theoretical specifications to be able to do so. Deductive geometric reasoning thus constitutes an important test case for psychological theories of human deduction and a privileged context to investigate intuitive deductive reasoning with mathematical content.

The aim of the present study is to identify and characterize the present bias in geometric reasoning, to pinpoint its cognitive origin, and to evaluate its implications for the psychology of mathematical thinking and deductive reasoning. We first asked whether the poor performance previously observed on I_3_ is an isolated phenomenon or the symptom of a larger bias. Our first experiment aimed at identifying biased inferences among a large set of geometric inferences with four premisses specifying relations between two points and two circles. This revealed a family of biased inferences but also showed that other geometric inferences with very similar properties were not affected. By comparing the geometric properties of the biased inferences with the non-biased ones, we observed that counterexamples to non-biased inferences can always be found by translating the circles in any possible representation of the premisses, while finding counterexamples to biased inferences may require scaling the circles. In a second experiment, we tested the hypothesis that inferences of the latter kind induce more deduction errors than those of the former kind. We reasoned further that biased inferences could be explained by the combination of systematic biases in both participants’ representations of the premisses and the kind of geometric transformations participants rely on when searching for counterexamples. Our third and fourth experiments addressed these two aspects directly. In our third experiment, we investigated in a drawing task how participants tend to represent the premisses of these geometric inferences. In our fourth experiment, we tested whether participants were more prone to rely on translation rather than scaling in searching for counterexamples.

## EXPERIMENT 1: IDENTIFYING THE BIASED INFERENCES

The geometric inferences I_2_ and I_3_ discussed above are very similar, and yet our previous work revealed that performance on I_3_ was much lower than on I_2_. To understand the reason behind this reasoning bias, our first step in this study was to obtain a more exhaustive picture of the geometric inferences that were affected by it. To this end, we generated algorithmically a large set of geometric inferences similar to I_2_ and I_3_ in the precise sense specified below. We then measure reasoning performance on all these inferences over 6 different groups of participants.

### Methods

#### Participants.

160 adults were recruited on the Amazon Mechanical Turk platform with the two following worker qualifications: “HIT Approval Rate (%) for all Requesters’ HITs greater than 95” and “Location is United States”.

#### Geometric Inferences.

We programmed an algorithm to systematically generate all possible geometric inferences involving two points and two circles, and where all relations but one between the four geometric objects were fixed in the premisses. We focused on the following set of topological relations: a point can be **inside**, **on**, or **outside** a circle; a circle can be **inside**, **outside**, or **intersect** another circle. The considered relations between the two circles correspond to all the possible relations between two distinct circles which do not involve tangency (we followed the characterization of intuitive deductive reasoning in Euclidean geometry advanced in Avigad et al., 2009; Manders, 2008). Thus, for the two circles *α* and *β*, the four possible relations were: “circle *α* is inside circle *β*”; “circle *β* is inside circle *α*”; “circle *α* intersects circle *β*”; and “circle *α* is outside circle *β*”. The algorithm proceeded by generating in a syntactic way all possible sets of four premisses where the relations between the objects ranged over the options just specified. Whenever an inference was generated, the algorithm checked whether or not it was already equivalent to an inference in the list of (non-equivalent) inferences to be kept. If the inference was equivalent to an inference in the list it was disregarded, otherwise it was added to the list. Two inferences were considered equivalent if one could be obtained from the other by reordering the premisses and/or renaming some of the objects in a different way (e.g., switching the names *α* and *β* for the two circles).

The program produced 60 invalid inferences, 39 valid inferences, and 36 inferences with a set of inconsistent premisses (i.e., where no geometric configuration in the Euclidean plane satisfies the premisses). We note that the invalid inferences can be divided into two categories depending on whether the conclusion consists of a relation between a point and a circle or between the two circles. The first category is composed of 42 inferences with 4 possible answers (see Table S1), where noticeably only 2 premisses out of 4 constrain the conclusion. The second category of invalid inferences is composed of 18 inferences with 5 possible answers.

#### Procedure.

All 60 invalid inferences were tested online, by presenting 160 adults randomly divided into 6 groups with 10 invalid inferences each. All groups were also presented with a common set of 10 valid inferences (Table S1). For each inference, after reading the premisses, participants were asked to choose, among a set of proposed conclusions, the one that necessarily followed, or to indicate that none of them followed (see Figure S1). For instance, participants may be told to consider a situation involving two points *A* and *B* and two circles *α* and *β* such that:

point *A*  is **inside**  circle *α*

point *A*  is **on**   circle *β*

point *B*  is **on**   circle *α*

point *B*  is **inside**  circle *β*

They were then asked to select from the proposed conclusions the one that necessarily follows, or to indicate that none of them follows:

circle *α*  is **inside**  circle *β*

circle *β*  is **inside**  circle *α*

circle *α*  **intersects**  circle *β*

circle *α*  is **outside**  circle *β*

 **none of the above follows** 

No diagrams were provided to participants in the reasoning task. Yet, given the difficulty of the task, they were allowed to use paper and pencil if they found it useful, and to spend as much time as they wanted on each inference. They were asked to always provide a response even if they were uncertain about the answer.

To ensure that all participants in the four experiments understood the definition of a circle and the meaning of the relations involved in the reasoning and drawing tasks, they had to successfully pass a geometry vocabulary test at the beginning of the session. The test consisted of seven questions covering all the relations involved in the tasks. In each question, a diagram was presented, and participants were asked to choose the correct statement describing the diagram. Three questions concerned the three possible relations between a point and a circle, the proposed answers were: “point *A* is inside circle *α*”, “point *A* is on circle *α*”, and “point *A* is outside circle *α*”. Four questions concerned the four possible relations between two circles, the proposed answers were: “circle *α* is inside circle *β*”, “circle *β* is inside circle *α*”, “circle *α* intersects circle *β*”, and “circle *α* is outside circle *β*” (see Figure S2). The order of the questions was randomized for each participant.

#### Determination of Sample Size.

In the absence, to our knowledge, of previous studies investigating similar questions, we arbitrarily set a sample size of 120 (20 participants per group). We thus first collected data in 120 participants, and then added participants by batches of 20 until we met the pre-determined sample size of usable data (see inclusion criteria below).

#### Inclusion Criteria.

To be allowed to participate in the online experiment, participants first had to pass the pre-study geometry vocabulary test just described (by answering correctly to at least six out of the seven questions). The whole task was challenging and required sustained concentration for about 30 minutes. As a consequence, a significant portion of participants indiscriminately answered as fast as possible without taking the time to examine each geometric situation. Those participants were eliminated from further analyses.

We excluded participants with a correct rate on valid inferences below 40% which corresponds to one standard deviation above chance level (at 25%) if participants’ answers followed a normal distribution. This criterion was applied only on valid inferences for two reasons. First, because only valid inferences were commonly presented to all participants. Second, because this inclusion criterion was determined on a subset of inferences that were independent of the inferences of interest.

Though we note that, by doing so, we arguably excluded participants exhibiting a response bias towards the answer “none of the above”, i.e., participants who were unsure of their response and performed poorly on valid inferences, but well on invalid inferences. To include those participants, they had to be distinguished from speeders who did not complete the task. We thus excluded participants who on average spent less than 15 seconds to answer. This was determined to be the minimum amount of time necessary to provide an answer to valid inferences with a correct rate of at least 60%. Only 12 participants whose responses were biased towards “none of the above” were found. We performed statistical analyses both after including or excluding those participants and found that the results remained virtually unchanged. The results reported here exclude those participants. Figure S3 reports the results obtained when including those participants.

#### Data Analysis.

Data from 123 participants (20 in groups 1 and 3, 17 in group 2, 21 in group 4 and 5, and 24 in group 6) were finally included in subsequent analyses, which were performed using R and the software RStudio. We used the *tidyverse* library to prepare the data, and Wilcoxon tests to analyze performance on each inference.

### Results and Discussion

On valid inferences, participants from all 6 groups performed similarly (group 1: 80.2 ± 4.99%; group 2: 79.7 ± 4.44%; group 3: 78.0 ± 4.14%; group 4: 78.1 ± 4.40%; group 5: 80.8 ± 3.58%; group 6: 72.7 ± 4.35%; *F*(1, 121) = 1.04, *p* = 0.311). This justified to pool all the groups together in subsequent analyses (accuracy range on 10 valid inferences: 68.3% to 86.2%; global % correct: 78.1 ± 1.76).

**Figure 1.**
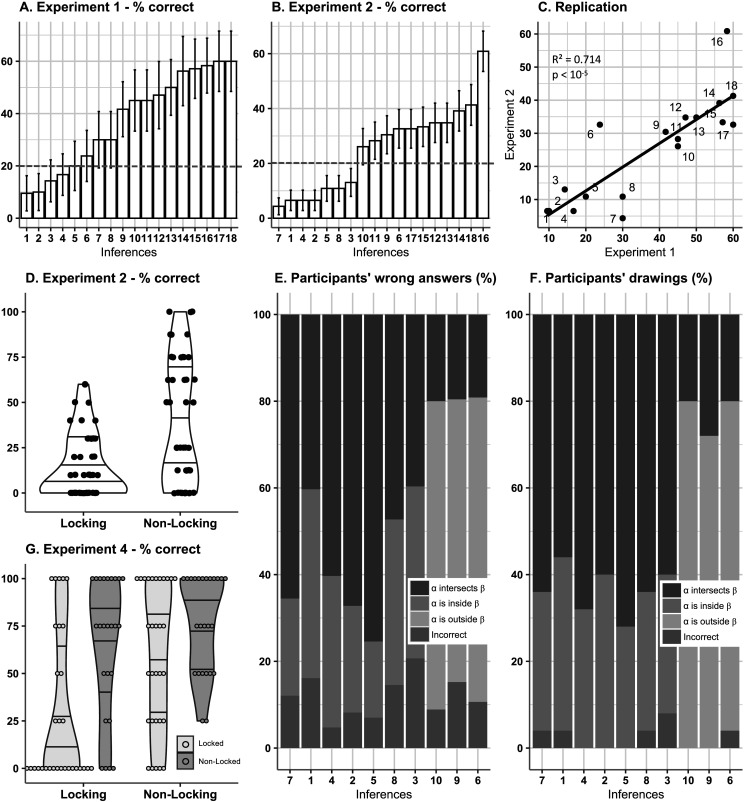
**Main results of the four experiments.** (A) shows the percent success averaged across participants for each invalid inference whose proposed conclusions concerned relations between the two circles, sorted in ascending order, observed in Experiment 1. The dotted line represents the chance level (here at 20%). (B) is the same as (A) for Experiment 2. (C) shows the correlation of performance level across inferences 1 to 18 between Experiments 1 and 2. (D) compares the distribution of percent success averaged across inferences with the locking property versus without the locking property, for all participants in Experiment 2. (E) shows the distribution of wrong conclusions for each inference with the locking property averaged across all participants from both experiments. (F) shows the distribution of topological configurations in the drawings produced in Experiment 3, i.e., in the representations of the premisses of each inference with the locking property produced by the participants. (G) compares the distribution of percent success averaged across inferences with the locking property versus without the locking property, and across the two diagram conditions locked configuration versus non-locked configuration.

For the invalid inferences, we found an overall performance of 59.7 ± 2.69% correct. Participants’ accuracy in determining that “none of the proposed conclusions followed” varied widely from 9.52% to 85.7%. Participants performed above chance on virtually all 42 invalid inferences whose conclusion established a relation between a point and a circle. For these inferences, the mean accuracy reached at least 42.9% and went up to 85.7% (all significantly above chance level of 25%: Wilcoxon *p*s < 0.05, except for the two inferences of lowest accuracy; global accuracy: 69.8 ± 2.98%). Participants’ overall accuracy on the 18 inferences whose conclusion concerned the relation between two circles only reached 35.4 ± 3.33% on average. Performance ranged from 9.52 ± 6.56% to 60.0 ± 11.2% and was significantly above chance (here at 20%; One-sided Wilcoxon *p*s < 0.05) for only six inferences (Figures 1A and S3A).

The results of Experiment 1 revealed that a whole set of geometric inferences are affected by the bias exhibited by inference I_3_, while many other similar inferences are not affected by it and in this respect are similar to I_2_. They also confirmed that adult participants from the general population are able to perform this geometric reasoning task as witnessed by their performances on valid inferences and non-biased invalid inferences. As we will now see, by examining the potential relations between the mathematical properties of these inferences and their associated reasoning performances one can generate cognitive hypotheses as to the origins of this reasoning bias.

### Interim Discussion: The Locking Property

**Figure 2.**
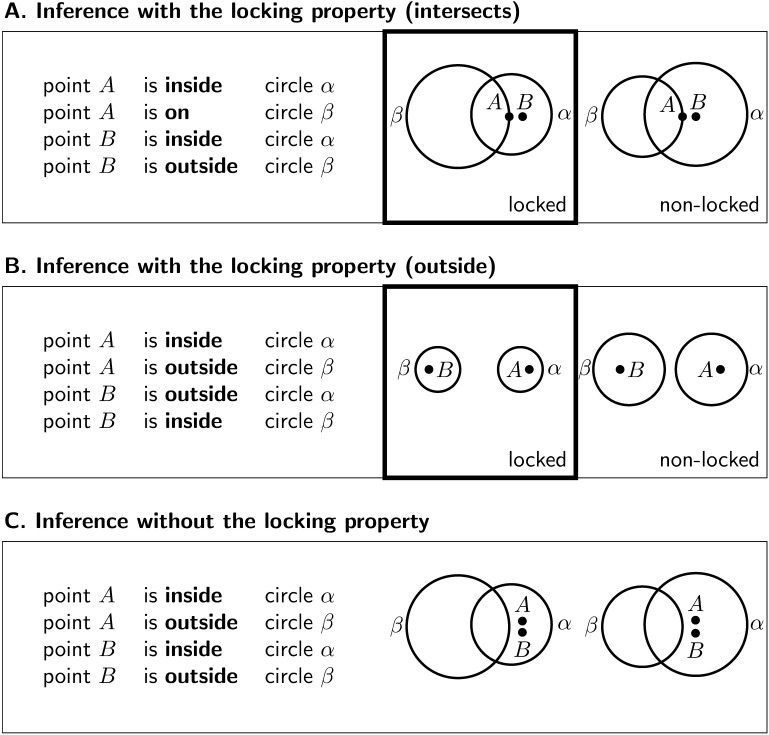
**Locking property.** (A, B) Examples of invalid inferences with the locking property. For locked configurations, it is not possible to reach an alternative topological configuration by merely translating the circles. A search procedure for alternative conclusions which starts from a locked configuration and only relies on translating the circles is thus going to miss alternative topological configurations; to find such alternatives, it is necessary to rely on scaling the circles. For non-locked configurations, it is always possible to reach an alternative configuration by translating the circles. (C) Example of an invalid inference without the locking property. Inferences without the locking property do not admit locked configuration. For any possible geometric representation of the premisses, it is always possible to find an alternative topological configuration by translating one of the circles.

What may explain the striking pattern of disparate performance observed on this set of invalid geometric inferences? Our previous work (Hamami et al., 2021) proposed that people judge a geometric inference as invalid when they have identified at least two different conclusions compatible with the premisses. To search for alternative conclusions, people would first generate a mental representation of a geometric configuration satisfying the premisses, and then apply geometric transformations on the objects present in the proposed conclusions. Now, only two kinds of elementary geometric transformations can be applied to circles: translation and scaling. For inferences 11 to 18 (Figures 1A and S4), we observed that an alternative configuration can always be found by only translating the circles in the initial configuration. However, that is not true for inferences 1 to 10 where some representations of the premisses are such that scaling the circles is necessary to find an alternative configuration. We say that an inference has the *locking property* whenever it admits *locked configurations*, i.e., geometric configurations where the relation between the two circles is preserved or stable under any possible translation of the circles (Figure 2)—locked configurations can be such that the two circles intersect (Figure 2A) or the two circles are disjoint (Figure 2B). For inferences with the locking property, if people start with a locked configuration and only rely on translating the circles when searching for alternative configurations, then their search procedure is doomed to fail—they will remain locked into the initial topological configuration (Figures 2A and 2B). In this case, the only way to find an alternative configuration is to scale the circles. By contrast, for inferences without the locking property, an alternative configuration can always be found by only translating the circles (Figure 2C). The locking property is then a geometric property, induced by the procedure just described, which divides the set of invalid inferences in two categories, and constitutes a good candidate for explaining why some of the reasoning problems appeared more difficult than others.

## EXPERIMENT 2: EFFECT OF THE LOCKING PROPERTY

We conducted a second experiment to specifically test the hypothesis that inferences with the locking property are more difficult than inferences without the locking property.

### Methods

#### Sample Size, Participants, and Inclusion Criteria.

Based on results from Experiment 1, we determined a sample size of at least 28 participants. Participants were, as in Experiment 1, adults recruited on the Amazon Mechanical Turk (AMT) platform with the two following worker qualifications: “HIT Approval Rate (%) for all Requesters’ HITs greater than 95” and “Location is United States”. Because of the presence of many speeders on the AMT platform, we decided to exceed our pre-determined sample size and recruited 88 participants. The inclusion criteria were exactly the same as for Experiment 1, leaving 46 participants.

#### Materials and Procedure.

Participants were presented with the same reasoning task as in Experiment 1, but this time focusing on the 18 invalid inferences of the second category (see Figure 1B, Figure S4, and Table S1), 10 with the locking property and 8 without. All 18 invalid inferences were presented together with 18 valid inferences over two online sessions (Table S1). The second session took place 5 days after the first one.

#### Data Analysis.

As in Experiment 1, we used the *tidyverse* library to prepare the data, and Wilcoxon tests to analyze performance on individual inferences. We also used standard *t*-tests when aggregating performance over multiple inferences, Chi-square tests to evaluate the distribution of wrong answers, and Pearson correlations to evaluate the replicability of results across experiments.

### Results and Discussion

Participants overall chose the correct option for valid inferences in 78.0 ± 2.41% of the cases, thus ensuring proper task completion. For invalid inferences, the profile of difficulty observed in Experiment 1 was strongly replicated (test of correlation between the correct rates observed in both experiments: Pearson *r* = 0.838, *R*^2^ = 0.702, *t*(16) = 6.15, *p* < 10^−4^, 95% CI = [0.61, 0.94]; see Figure 1C). We again observed very disparate performances, the accuracy for each inference ranging from 4.35 ± 3.04% to 60.9 ± 7.28% (Figure 1B). Participants’ overall accuracy on invalid inferences was 25.2 ± 3.50%. Within those invalid inferences, we found that inferences with the locking property were more difficult than inferences without the locking property. Participants’ mean accuracy on inferences with the locking property only equaled 14.8 ± 2.67%, while they reached 38.3 ± 5.09% of correct responses on inferences without the locking property (paired *t*-test: *t*(45) = 6.59, *p* < 10^−7^, 95% CI = [16.3, 30.7], Cohen’s *d* = 0.97, see Figure 1D). We note that within inferences with the locking property, performance on inferences 1, 2, 3, 4, 5, 7, and 8 which were systematically mistaken as valid (the accuracy in the reasoning task is significantly below chance level: all Wilcoxon *p*s < 0.001) was lower than performance on inferences 6, 9, and 10.

We reasoned further that the wrong answer participants selected was not only indicating their failure to detect invalid inferences but was also likely reflecting their initial mental representation of the geometric situation, i.e., the topological configuration they were locked into. For inferences with the locking property, we thus expected to find that the dominant wrong answer corresponded to the locked configurations, and that is exactly what we observed (Figure 1E). Pooling all participants from both experiments together, we indeed found that the dominant wrong answer for inferences 1, 2, 3, 4, 5, 7, and 8 was “circle *α* intersects circle *β*” (Chi-Square test: 10^−10^ < *p*s < 0.008, effect sizes: 0.34 < *w*s < 0.86; except for inferences 1, 3, and 8), which corresponds to the locked configurations. And for inferences 6, 9, and 10, the locked configurations are such that circle *α* is outside circle *β*, which also corresponds to the dominant wrong answer in these cases (Chi-Square test: all *p*s < 10^−7^, effect sizes: 0.86 < w*s* < 0.88).

The results of Experiment 2 confirmed that inferences with the locking property are more difficult than inferences without the locking property, thereby replicating the results of Experiment 1 in a different group of adults. They also revealed that participants’ dominant wrong answers turn out to be of the same type as locked configurations. Poor performance on inferences with the locking property may then be explained by the combination of two cognitive biases, namely (1) a tendency to produce locked configurations when representing the premisses, and (2) a tendency to rely on translating but not scaling the circles when searching for alternative configurations. We tested the two parts of this explanation in two dedicated experiments.

## EXPERIMENT 3: PREFERRED CONFIGURATIONS IN REPRESENTING THE PREMISSES OF INFERENCES WITH THE LOCKING PROPERTY

The first part of the above explanation proposes that people got locked on unwarranted conclusions because they tend to produce locked configurations when representing the premisses of inferences with the locking property. To test this hypothesis, we asked another group of participants to draw a representation of the geometric situation described by the premisses of inferences 1 to 18.

### Methods

#### Participants.

Participants were 28 undergraduate students at Harvard University who took part in the study in person and obtained course credits at the Department of Psychology for their participation.

#### Materials and Procedure.

Participants were provided with a test booklet and a pencil. On each page of the booklet, they were asked to represent the situation described by the set of four premisses composing each of the 18 invalid inferences tested in Experiment 2. Participants were simply instructed to draw each situation freehand as carefully as possible and to report the names of the objects on the drawing.

#### Sample Size Determination and Inclusion Criteria.

Given the difficulty to test participants in person directly after COVID-19 restrictions were lifted at the University, given temporal constraints implied by the obtention of course credits, and in the absence of preliminary data testing such drawing preferences, we aimed at collecting data in at least 22 participants (which would be enough to detect large effects in a Chi-square test with 1 degree of freedom, a significance level of 0.05, and a standard power of 0.8). 28 students volunteered to participate in this study. 3 participants misinterpreted the instructions and drew a representation of each premise instead of one representation of the situation described by all four premisses together. Data from these 3 participants were thus excluded, leaving data from 25 participants finally included in the subsequent analysis.

### Results and Discussion

Figure 1F displays the preferred configurations drawn by participants for all 10 inferences with the locking property. We found that for inferences 1, 2, 3, 4, 5, 7, and 8, participants preferentially drew a configuration in which circle *α* intersects circle *β* (in at least 60% of the cases, Chi-Square test: 10^−5^ < *p*s < 0.05, effect sizes: 0.40 < w*s* < 0.88, except for inference 1: *p* = 0.096, *w* = 0.33). For inferences 6, 9, and 10, participants preferentially drew a configuration in which circle *α* is outside circle *β* (in at least 72% of the cases, Chi-Square test: 10^−9^ < *p*s < 10^−4^, effect sizes: 0.88 < w*s* < 1.20). These results are strikingly similar to the analysis of wrong answers reported in Experiments 1 and 2 (see Figures 1E and 1F). For inferences 1, 2, 3, 4, 5, 7, and 8, the preferential configurations were overwhelmingly locked (90.1% of the cases), thus suggesting that people tend to produce locked configurations when representing the premisses of inferences with the locking property that are systemically misjudged as valid. For inferences 6, 9, and 10, participants’ preferential configurations were locked in 15.8% of the cases. If this latter result seems at first sight to contrast sharply with the former one, it is in fact fully coherent with participants’ higher performance for inferences 6, 9, and 10 than for inferences 1, 2, 3, 4, 5, 7 and 8 in Experiments 1 and 2. These results altogether suggest that people’s reasoning performance can be related to their tendency to produce locked configurations.

## EXPERIMENT 4: REASONING FROM LOCKED VS NON-LOCKED CONFIGURATIONS—SCALING VS TRANSLATING IN SEARCHING FOR ALTERNATIVE CONCLUSIONS

The second part of the explanation proposes that people got locked on unwarranted conclusions for inferences with the locking property because they are more likely to rely on translating rather than scaling the circles when searching for alternative configurations. This can be tested by exploiting the fact that inferences with the locking property admit both locked and non-locked configurations. If people are more likely to rely on translating than scaling the circles, then they will be more likely to find an alternative configuration if they start with a non-locked configuration as opposed to a locked configuration. In this fourth experiment, we tested this hypothesis by asking participants to evaluate inferences, as in Experiments 1 and 2, but this time we also provided them with a diagram consisting of one possible representation of the premisses (see Figure S5). Inferences with the locking property were presented with locked configurations in one condition and with non-locked configurations in the other.

### Methods

#### Participants.

81 undergraduate students at Harvard University participated in this online experiment. They were awarded course credits at the Department of Psychology for their participation.

#### Materials and Procedure.

In this experiment, we used a subset of the inferences tested in Experiment 2. We picked 4 invalid inferences with the locking property (invalid inferences 1, 2, 4, and 5 in Figure S4 and Table S1) and 4 inferences without the locking property (invalid inferences 12, 13, 14, and 17 in Figure S4 and Table S1), together with 8 valid inferences (valid inferences 1, 2, 3, 4, 5, 6, 7, and 8 in Table S1).

**Figure 3.**
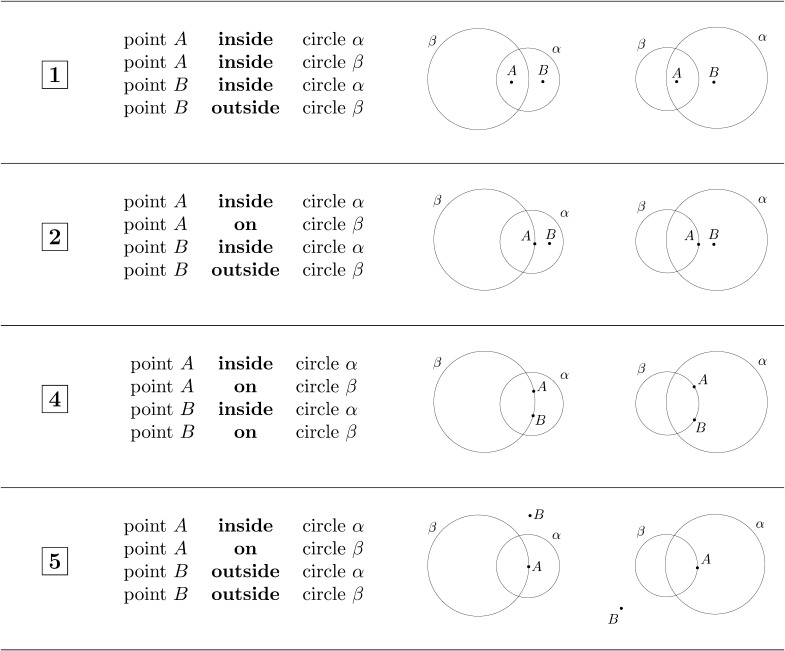
Invalid inferences with the locking property tested in Experiment 4 together with the diagrams provided in the two conditions. Participants assigned to the locked condition (group 1) saw the inferences together with the diagrams of the left column. Participants assigned to the non-locked condition (group 2) saw the inferences together with the diagrams of the right column. In the locked (resp. non-locked) condition, diagrams for invalid inferences with the locking property consisted of locked (resp. non-locked) configurations.

Participants were presented with a similar reasoning task as in Experiments 1 and 2, but this time, each inference was accompanied with a diagram depicting one possible representation of its premisses (see Figure S5 for a screenshot of the task). Participants were randomly assigned to two possible conditions. In one condition, the diagrams provided for inferences with the locking property consisted of locked configurations, and in the other condition they consisted of non-locked configurations (see Figure 3). For the locked configurations, circle *β* was bigger than circle *α* with a ratio of 1.6:1; for the non-locked configurations, it was the other way around. As can be seen in Figure 3, it is not possible to reach a counterexample by only translating the circles for the locked configurations, while it is possible to do so for the non-locked configurations. The diagrams for valid inferences and for invalid inferences without the locking property were designed to be as similar as possible to the diagrams for inferences with the locking property (see Figure S6 for all the diagrams used in the two conditions). In particular, the two circles were always positioned in the exact same way, only the positions of points *A* and *B* differ in each diagram according to the premisses.

#### Sample Size Determination and Inclusion Criteria.

The sample size corresponding to detecting large effects in a two-sample Wilcoxon test with a significance level of 0.05 and a standard power of 0.8, is at least 50. We thus opened 100 online testing slots on the Harvard Study Pool. 81 students signed up to participate in our study. Two of the easiest inferences—invalid inference 16 (Figure S4) and valid inference 9 (Table S1) were presented without diagrams as part of the instructions. To ensure that all participants included in our analysis understood the task, data from participants who did not provide the correct answer to either of these inferences were excluded. A total of 56 participants were finally included, 31 in the locked condition (group 1), and 25 in the non-locked condition (group 2).

### Results and Discussion

We first verified that both groups of participants’ performed similarly on valid inferences (group 1: 91.1 ± 2.73%, group 2: 90.0 ± 3.15%; *t*(54) = 0.27, *p* = 0.787), thus justifying comparing both groups on invalid inferences in the following. For inferences without the locking property, participants from the first group answered correctly in 59.7 ± 6.83% of the cases, and participants from the second group answered correctly in 77.0 ± 5.19% of the cases—a difference that did not reach significance level (*w* = 292, *p* = 0.10, *z* = 1.65, EF = 0.22; average on both groups: 67.4%; Figure 1G). For invalid inferences with the locking property, we found that participants from group 2 who were presented with a non-locked configuration performed significantly better than participants from group 1 who were presented with a locked configuration (group 1: 29.0 ± 7.16%, group 2: 65.0 ± 7.36%, *w* = 206, *p* = 0.002, *z* = 3.12, EF = 0.42; Figure 1G). The type of configuration (locked/non-locked) also marginally interacted with the type of invalid inference (*w* = 277, *p* = 0.06, *z* = 1.9, EF = 0.25; Figure 1G).

In sum, participants were less likely to find alternative configurations to an invalid inference with the locking property when they were presented with a locked configuration of the premisses than when they were presented with a non-locked configuration. This suggests that people are more likely to rely on translating rather than scaling the circles when searching for alternative configurations. Experiment 4 thus provides evidence for the second part of our explanation, namely that the geometric reasoning bias reported here is in part due to a bias with respect to the geometric transformations recruited in the counterexample search process.

## GENERAL DISCUSSION

In this study, we have identified a robust cognitive bias in deductive geometric reasoning, an area that has so far received little attention in mathematical cognition and the psychology of reasoning. Our results show that people systematically misjudge as valid a particular set of invalid geometric inferences while performing significantly better on another set of invalid inferences with very similar characteristics. An examination of the geometric properties of the two sets of inferences revealed that, for the inferences with the highest level of performance, it is always possible to find a counterexample by simply translating the circles when starting from any geometric representation of the premisses. This is not the case for the inferences with the lowest level of performance which, depending on the geometric representation one is starting with, may require to rely on scaling the circles to reach a counterexample. We refer to this distinction as the locking property, a geometric property that divides our geometric inferences in two categories. Our second experiment showed that the difference in reasoning performance between inferences with and without the locking property was highly significant. It also showed that the dominant wrong answers for inferences with the locking property corresponded to the geometric configurations that require scaling the circles to reach a counterexample—what we called locked configurations. Experiments 3 and 4 further investigated the origin of this bias by addressing separately the representation of the premisses and the reasoning from a given geometric representation. Experiment 3 showed that people have indeed a preference to produce locked configurations when asked to represent the premisses of inferences with the locking property that are systemically misjudged as valid. Experiment 4 showed that people are more likely to judge an inference with the locking property as valid when they are provided with a locked configuration as opposed to a non-locked configuration. In fact, performance in this latter case turned out to be almost similar to performance on inferences without the locking property. This means that it is possible to eliminate, or at least drastically diminish, the bias by manipulating the representation of the premisses provided. Taken together, the results of Experiments 3 and 4 provide evidence that the cognitive bias reported here is due to the combination of two biases in the way people represent a situation described in a set of geometric propositions and in the way counterexample search proceeds on a given geometric representation of the premisses.

A simple explanation of this reasoning bias would be that participants impose the extra constraint that the circles must be of the same size. This would straightforwardly explain why participants would conclude that the two circles must intersect for inferences with the locking property of type “intersects” (Figure 2A). For, in these cases, the only alternative configuration always involves one circle being inside the other, which is impossible if the circles are of the same size. However, by the same tenet, we should also have observed poor performance on inferences 12 and 13 since the only alternative configurations to the two circles intersecting in these cases are such that one circle must be inside the other. Experiment 4 provides further evidence against this explanation. In this experiment, we provided participants with geometric representations where the circles were clearly not of the same size, thus ruling out the possible interpretation that the circles have to be of the same size. And yet, we observed again the same pattern of performance between inferences with and without the locking property. Another simple explanation would be that people do not engage in counterexample search at all and simply pick the conclusion depicted in their initial representation of the premisses. This would readily explain the biased inferences, for if people only consider one model of the premisses, then they would misjudge any of our invalid inferences as valid. But this could not explain the overall difference in performance between locking and non-locking inferences, for in the two cases participants should have misjudged them as valid.

The current psychological theories of human deductive reasoning, even when concerned with spatial reasoning, do not seem equipped to predict the difference in difficulty between geometric inferences with and without the locking property. In the mental model theory (Johnson-Laird, 2010), the difficulty of an inference is predicted by the number of mental models compatible with the premisses. According to the most natural interpretation of what a mental model is in the present geometric context (Figure S4), inferences 1 to 10 have 2 mental models while inferences 11 to 18 have 3 mental models, with the exception of inference 16 which has 4 mental models (Figure S4). Contrary to what the mental model theory predicts, we observed that inferences with 2 mental models (the inferences with the locking property) were more difficult than inferences with 3 and 4 mental models (the inferences without the locking property) (see Figures 1A, 1B, and 1D). The profile of difficulty cannot be explained either in terms of distance to the closest countermodel(s) (Ragni & Knauff, 2013) since, for the 18 invalid inferences we consider, any alternative model to the initial one is already a countermodel. According to rule-based theories of reasoning (Rips, 1994), an invalid inference can be misjudged as valid if one has either applied an incorrect inference rule or has misapplied a correct one. However, such an approach would only be ad-hoc unless an additional explanation is provided as to why people are more likely to apply incorrect rules, or misapplied correct ones, for some inferences but not others. Probabilistic models of geometric reasoning could be developed in the spirit of the new paradigm (Oaksford & Chater, 2020) or in terms of simulation-based statistical models (Hart et al., 2018). But it remains to be seen how much geometry should be integrated into a probabilistic model in order to predict the cognitive phenomena reported here. From the perspective of dual-process theories (Evans, 2008; Kahneman, 2011), the poor performance on inferences with the locking property may be interpreted as a failure to overcome an initial intuitive incorrect answer by reflection. The situation here would then be similar to what has been observed in the Cognitive Reflection Test (Frederick, 2005) where an initial incorrect answer comes to mind readily which can then be overridden by reflection. However, it is hard to see why participants would fail to engage in a reflection process for inferences with the locking property while succeeding in doing so for inferences without the locking property since both types of inferences have the exact same form.

The systematic reasoning errors reported in the present study can be explained by hypothesizing a reasoning procedure which operates on geometric configurations and relies on geometric transformations (Hamami et al., 2021). If people’s reasoning (1) starts with a mental representation of the premisses which is a locked configuration, and (2) only appeal to processes that consist in translating the circles and/or scaling them in a limited range, then they will most likely miss alternative conclusions for invalid inferences with the locking property. In addition, as observed in Experiments 1 and 2, they will likely conclude that “circle *α* intersects circle *β*” (resp. that “circle *α* is outside circle *β*”) for the inferences where the locked configurations are such that circle *α* intersects circle *β* (resp. circle *α* is outside circle *β*). Experiments 3 and 4 provide direct evidence in support of (1) and (2). Similarly to previous findings on preferences in representing spatial arrangements (see, e.g., Ragni & Knauff, 2013), participants in our Experiment 3 exhibited clear preferences for certain representations of the premisses. For inferences with the locking property inducing systematic reasoning errors, these preferential representations were overwhelmingly locked. In our Experiment 4, participants more likely misjudged an inference with the locking property as valid when they were provided with a configuration which required to scale the circles to find a counterexample. This result suggests that people do not rely on scaling but on translating the circles in this reasoning task. This could reflect a general tendency in human’s reasoning, as similar results have previously been found in other contexts. For example, participants were faster to solve the Tower of Hanoi problem when the rules involved translating the pieces than when they involved scaling them (Kotovsky et al., 1985). This general tendency might come from the fact that, similarly to the so-called “insight problems” (Gilhooly & Murphy, 2005) such as the nine dot problem, people would bring extra assumptions or constraints in their initial interpretation of the problem—e.g., in the nine dots problem, people impose the additional constraint that you cannot draw lines outside the square formed by the dots. For inferences with the locking property, people may have imposed the extra assumption that the circles have to be rigid objects, and so that it is not allowed to scale them. To identify that these inferences are invalid, people would then need to reframe their initial representation either by lifting this assumption or by producing another representation of the premisses that is not a locked configuration.

Further work is required to determine whether the failure to rely on scaling is to be found in human spontaneous geometric representations themselves, or in the way these representations are recruited by higher-level cognitive processes. This could provide key insights on how our core knowledge of geometry transitions into higher-level cognitive abilities such as deductive geometric reasoning (Newcombe et al., 2019; Spelke et al., 2010; Ullman & Tenenbaum, 2020). Given that geometry is the privileged subject by which young students are introduced to mathematics and deductive reasoning (Ferrini-Mundy, 2000; Koedinger, 1991), understanding the source of such cognitive biases in deductive geometric reasoning may also prove useful to education by identifying obstacles in the passage from intuitive to school mathematics where deduction takes the central stage (Dillon et al., 2017; Hawes et al., 2022). Pursuing research in this direction may provide further insights on some of the cognitive abilities essential to one of the major intellectual achievements of Western culture, namely the emergence of Euclidean geometry as a deductive theory of space.

## METHODS COMMON TO ALL EXPERIMENTS

### Ethics Statement

All participants provided informed consent prior to the experiments and were compensated for their participation. The experiments were performed according to the Declaration of Helsinki (2013) and approved by the Harvard University Institutional Review Board.

### Transparency and Openness

We report how we determined our sample size, all data exclusions, all manipulations, and all measures in the study. This set of studies was not preregistered, but all data and the code behind these results have been made publicly available at OSF and can be accessed at https://osf.io/j46h3/. Data were analyzed using R, version 4.2.3, and the packages *tidiyerse*, *rcompanion*, *lsr*, and *ggplot*.

## ACKNOWLEDGMENTS

The authors would like to thank Gia-Han Le for helping with data collection, and Wendy Erselius for her support with the Harvard Study Pool.

## FUNDING INFORMATION

This work was supported by the Centre for Logic and Philosophy of Science, Vrije Universiteit Brussel, by a postdoctoral research fellowship from the F.R.S.-FNRS to Y.H., and by the European Union’s Horizon Europe research and innovative programme under the Marie Sklodowska-Curie grant agreements No. 101063894 to Y.H. and No. 839611 to M.A.

## AUTHOR CONTRIBUTIONS

Y.H.: Conceptualization; Data curation; Funding acquisition; Investigation; Methodology; Validation; Visualization; Writing – original draft; Writing – review & editing. M.A.: Conceptualization; Data curation; Formal analysis; Funding acquisition; Methodology; Validation; Visualization; Writing – original draft; Writing – review & editing.

## DATA AVAILABILITY STATEMENT

This set of studies was not preregistered. All data and the code behind these results analysis have been made publicly available at OSF and can be accessed at https://osf.io/j46h3/.

## Note

^1^ Note that throughout, inferences are presented using the premisses-line-conclusion format with premisses and conclusion respectively written above and below the conclusion line.

## Supplementary Material



## References

[bib1] Amalric, M., Wang, L., Pica, P., Figueira, S., Sigman, M., & Dehaene, S. (2017). The language of geometry: Fast comprehension of geometrical primitives and rules in human adults and preschoolers. PLOS Computational Biology, 13(1), e1005273. 10.1371/journal.pcbi.1005273, 28125595 PMC5305265

[bib2] Avigad, J., Dean, E., & Mumma, J. (2009). A formal system for Euclid’s *Elements*. The Review of Symbolic Logic, 2(4), 700–768. 10.1017/S1755020309990098

[bib3] Dehaene, S., Izard, V., Pica, P., & Spelke, E. (2006). Core knowledge of geometry in an Amazonian indigene group. Science, 311(5759), 381–384. 10.1126/science.1121739, 16424341

[bib4] Dillon, M. R., Huang, Y., & Spelke, E. S. (2013). Core foundations of abstract geometry. Proceedings of the National Academy of Sciences, 110(35), 14191–14195. 10.1073/pnas.1312640110, 23940342 PMC3761620

[bib5] Dillon, M. R., Kannan, H., Dean, J. T., Spelke, E. S., & Duflo, E. (2017). Cognitive science in the field: A preschool intervention durably enhances intuitive but not formal mathematics. Science, 357(6346), 47–55. 10.1126/science.aal4724, 28684518

[bib6] Euclid. (1959). Elements. In Euclid’s Elements: All thirteen books complete in one volume. Dover Books. https://hdl.handle.net/2027/nyp.33433069105918

[bib7] Evans, J. S. B. T. (1989). Bias in human reasoning: Causes and consequences. Lawrence Erlbaum Associates, Inc.

[bib8] Evans, J. S. B. T. (2008). Dual-processing accounts of reasoning, judgment, and social cognition. Annual Review of Psychology, 59, 255–278. 10.1146/annurev.psych.59.103006.093629, 18154502

[bib9] Ferrini-Mundy, J. (2000). Principles and standards for school mathematics: A guide for mathematicians. Notices of the American Mathemathical Society, 47(8), 868–876.

[bib10] Frederick, S. (2005). Cognitive reflection and decision making. Journal of Economic Perspectives, 19(4), 25–42. 10.1257/089533005775196732

[bib11] Gilhooly, K. J., & Murphy, P. (2005). Differentiating insight from non-insight problems. Thinking & Reasoning, 11(3), 279–302. 10.1080/13546780442000187

[bib12] Hamami, Y., Mumma, J., & Amalric, M. (2021). Counterexample search in diagram-based geometric reasoning. Cognitive Science, 45(4), e12959. 10.1111/cogs.12959, 33873252

[bib13] Hart, Y., Dillon, M. R., Marantan, A., Cardenas, A. L., Spelke, E., & Mahadevan, L. (2018). The statistical shape of geometric reasoning. Scientific Reports, 8, 12906. 10.1038/s41598-018-30314-y, 30150653 PMC6110727

[bib14] Hartshorne, R. (2000). Geometry: Euclid and beyond. Springer. 10.1007/978-0-387-22676-7

[bib15] Hawes, Z. C. K., Gilligan-Lee, K. A., & Mix, K. S. (2022). Effects of spatial training on mathematics performance: A meta-analysis. Developmental Psychology, 58(1), 112–137. 10.1037/dev0001281, 35073120

[bib16] Izard, V., Pica, P., Spelke, E. S., & Dehaene, S. (2011). Flexible intuitions of Euclidean geometry in an Amazonian indigene group. Proceedings of the National Academy of Sciences, 108(24), 9782–9787. 10.1073/pnas.1016686108, 21606377 PMC3116380

[bib17] Johnson-Laird, P. N. (2010). Mental models and human reasoning. Proceedings of the National Academy of Sciences, 107(43), 18243–18250. 10.1073/pnas.1012933107, 20956326 PMC2972923

[bib18] Kahneman, D. (2011). Thinking, fast and slow. Farrar, Straus and Giroux.

[bib19] Kant, I. (1787). Critique of pure reason. P. Guyer & A. W. Wood (Eds.). Cambridge University Press. 10.1017/CBO9780511804649

[bib20] Kline, M. (1972). Mathematical thought from ancient to modern times. Oxford University Press.

[bib21] Koedinger, K. R. (1991). Tutoring concepts, percepts, and rules in geometry problem solving [Unpublished doctoral dissertation]. Psychology Department, Carnegie Mellon University.

[bib22] Kotovsky, K., Hayes, J. R., & Simon, H. A. (1985). Why are some problems hard? Evidence from Tower of Hanoi. Cognitive Psychology, 17(2), 248–294. 10.1016/0010-0285(85)90009-X

[bib23] Manders, K. (2008). The Euclidean diagram (1995). In P. Mancosu (Ed.), The philosophy of mathematical practice (pp. 80–133). Oxford University Press. 10.1093/acprof:oso/9780199296453.003.0005

[bib24] Netz, R. (1999). The shaping of deduction in Greek mathematics: A study in cognitive history. Cambridge University Press. 10.1017/CBO9780511543296

[bib25] Newcombe, N. S., Booth, J. L., & Gunderson, E. A. (2019). Spatial skills, reasoning, and mathematics. In J. Dunlosky & K. A. Rawson (Eds.), The Cambridge handbook of cognition and education (1st ed., pp. 100–123). Cambridge University Press. 10.1017/9781108235631.006

[bib26] Oaksford, M., & Chater, N. (2020). New paradigms in the psychology of reasoning. Annual Review of Psychology, 71, 305–330. 10.1146/annurev-psych-010419-051132, 31514580

[bib27] Plato. (1961). Meno. In E. Hamilton & H. Cairns (Eds.), The collected Dialogues. Princeton University Press. https://classics.mit.edu/Plato/meno.html

[bib28] Ragni, M., & Knauff, M. (2013). A theory and a computational model of spatial reasoning with preferred mental models. Psychological Review, 120(3), 561–588. 10.1037/a0032460, 23750832

[bib29] Rips, L. J. (1994). The psychology of proof: Deductive reasoning in human thinking. MIT Press. 10.7551/mitpress/5680.001.0001

[bib30] Spelke, E., Lee, S. A., & Izard, V. (2010). Beyond core knowledge: Natural geometry. Cognitive Science, 34(5), 863–884. 10.1111/j.1551-6709.2010.01110.x, 20625445 PMC2897178

[bib31] Tversky, A., & Kahneman, D. (1974). Judgment under uncertainty: Heuristics and biases. Science, 185(4157), 1124–1131. 10.1126/science.185.4157.1124, 17835457

[bib32] Ullman, T. D., & Tenenbaum, J. B. (2020). Bayesian models of conceptual development: Learning as building models of the world. Annual Review of Developmental Psychology, 2, 533–558. 10.1146/annurev-devpsych-121318-084833

