# Supplementary material for: Going Round in Circles: A Cognitive Bias in Geometric Reasoning
Source: Open Mind (Camb). 2024 Nov 22;8:1312–29. doi: 10.1162/opmi_a_00169 (PMC11627530; doi:10.1162/opmi_a_00169)
Supplement: Supplementary file 1 [file opmi-08-1312-s001.pdf]

Supplementary Materials

A Cognitive Bias in Geometric Reasoning

Yacin Hamami

Department of Philosophy, Université de Liège

Department of Humanities, Social and Political Sciences, ETH Zürich

Institut Jean Nicod, Department of Cognitive Studies, ENS, EHESS, PSL University, CNRS

Marie Amalric

Laboratory for Developmental Studies, Department of Psychology, Harvard University

Center for Brain/Mind Science (CIMeC), Universita degli studi di Trento

Author Note

Corresponding authors:

Yacin Hamami, 7 Place du 20 Août, 4000 Liège, Belgium,

Email: yacin.hamami@gmail.com.

Marie Amalric, 5000 Forbes Ave, Pittsburgh, PA 15213, USA,

Email: marie.amalric@normalesup.org.

Figure S1. A screenshot of the reasoning task with an example of a geometric inference to be evaluated. Participants were presented with four premisses describing the relations between two points and two circles. They were then asked to indicate whether one of the proposed conclusions followed, or that none of them followed, in a multiple-choice format.

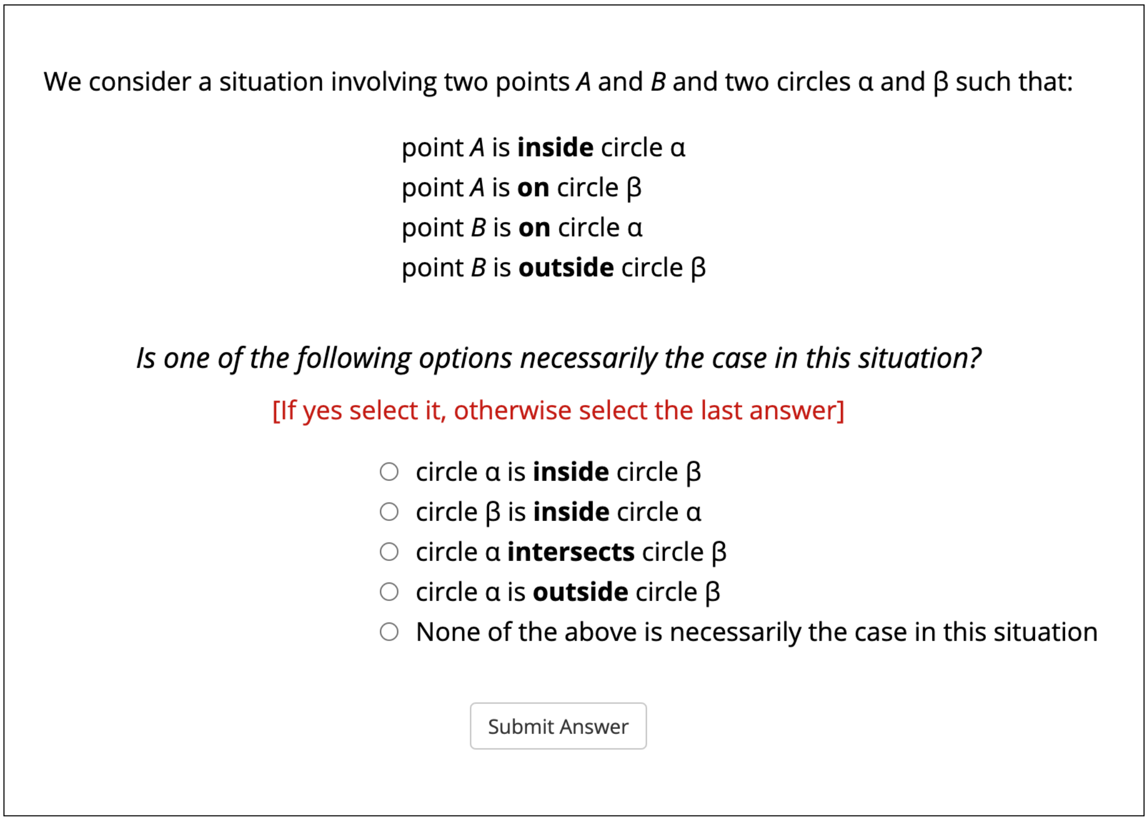

Figure S2. The seven questions included in the Geometry Vocabulary Test. This short test is presented at the beginning of each Experiment, to ensure participants' understanding of the definitions of a circle and of the relations on, inside, outside, and intersect used in the Experiments. The three questions at the top present the three possible relations between a point and a circle, and the four questions at the bottom, the four possible relations between two circles. The seven questions are presented in random order.

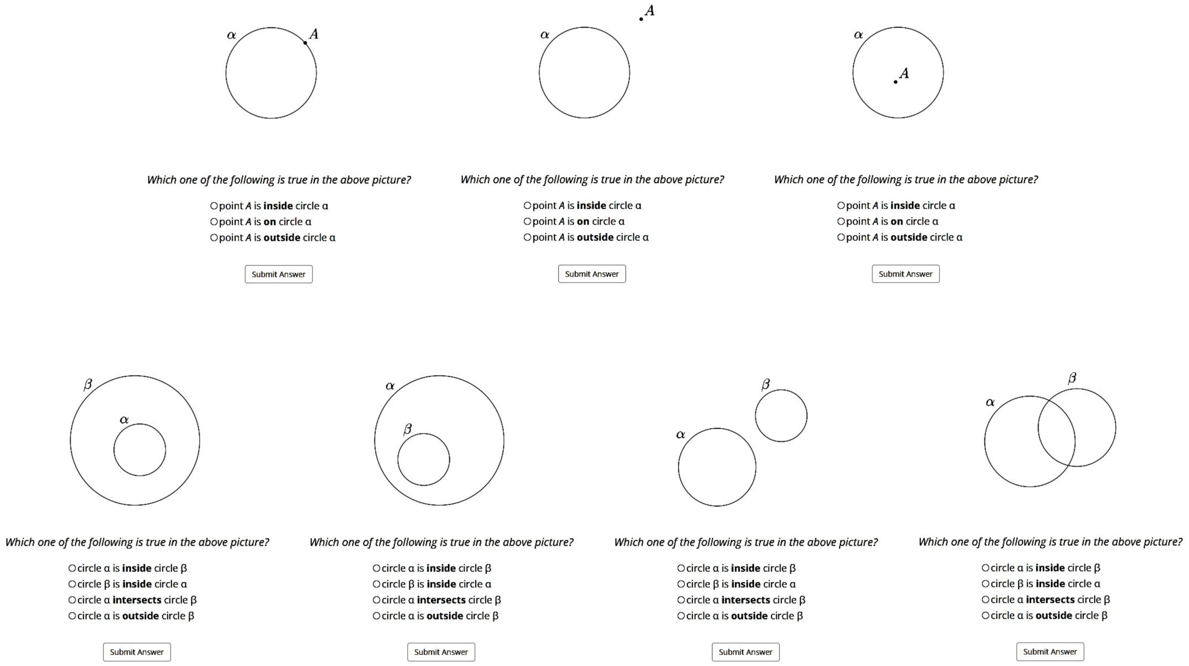

Figure S3. These are the same graphs A, B, C, D and E as in Figure 1 but for the case where participants with a response bias towards the answer “none of the above” were included. In this case, data from 135 participants in Experiment 1 and 49 participants in Experiment 2 were analyzed. The main results with this inclusion criteria remained virtually unchanged.

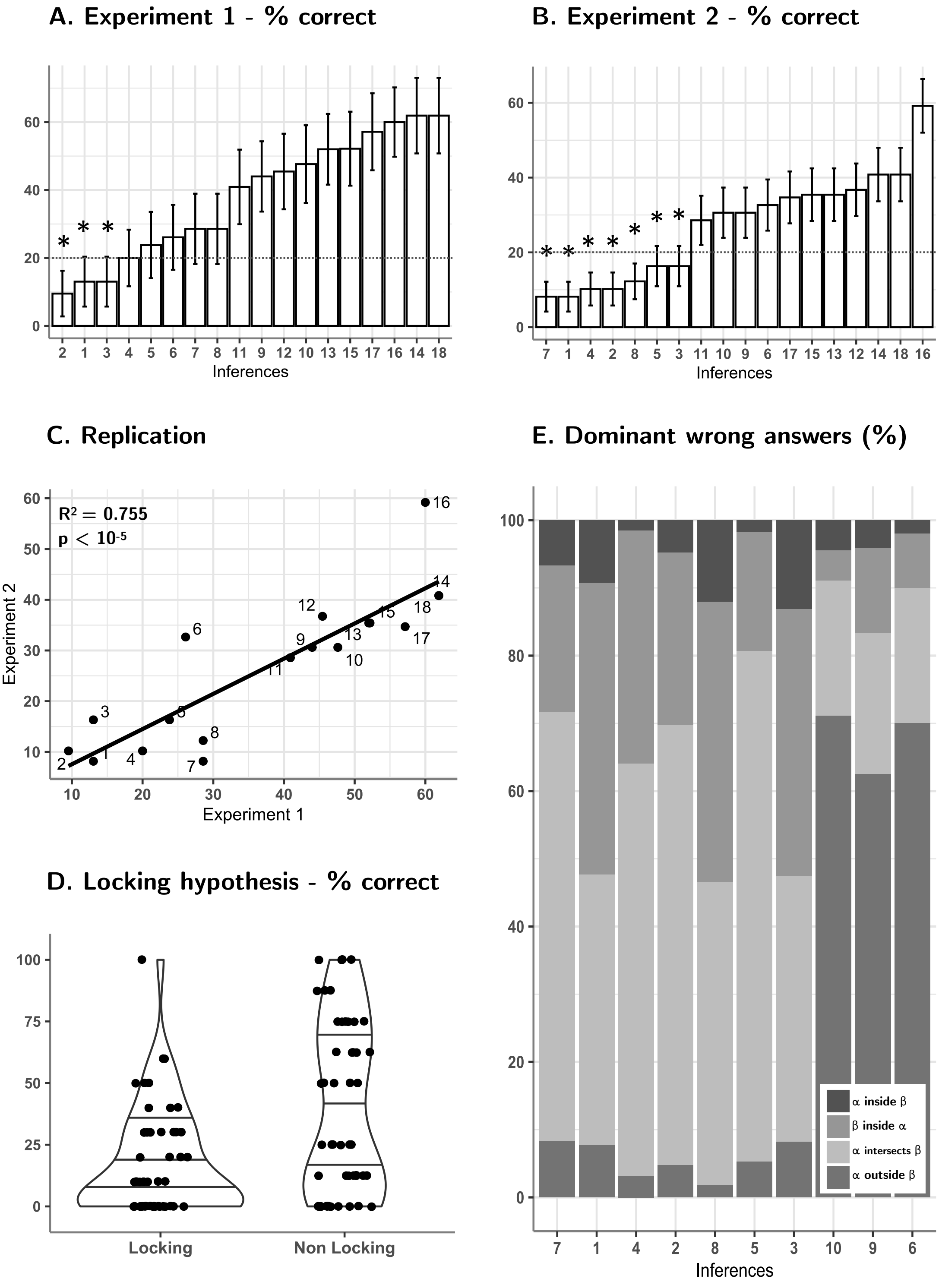

Figure S4. The 18 invalid inferences tested in Experiment 2. We represent the premisses together with geometric configurations corresponding to the conclusions compatible with the premisses—the proposed conclusions were "circle α is inside circle β", "circle β is inside circle α", "circle α intersects circle β", and "circle α is outside circle β". Inferences 1 to 10 have the locking property, while inferences 11 to 18 do not have the locking property.

1

point A	**inside**	circle α
point A	**inside**	circle β
point B	**inside**	circle α
point B	**outside**	circle β

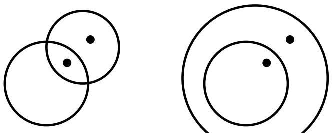

2

point A	**inside**	circle α
point A	**on**	circle β
point B	**inside**	circle α
point B	**outside**	circle β

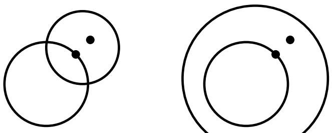

3

point A	**inside**	circle α
point A	**inside**	circle β
point B	**on**	circle α
point B	**outside**	circle β

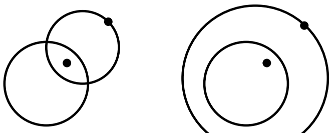

4

point A	**inside**	circle α
point A	**on**	circle β
point B	**inside**	circle α
point B	**on**	circle β

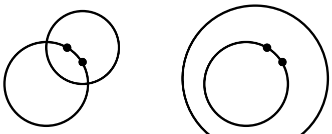

5

point A	**inside**	circle α
point A	**on**	circle β
point B	**outside**	circle α
point B	**outside**	circle β

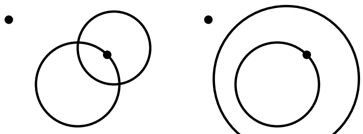

6

point A	**inside**	circle α
point A	**outside**	circle β
point B	**outside**	circle α
point B	**inside**	circle β

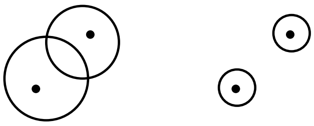

7

point A	**inside**	circle α
point A	**on**	circle β
point B	**on**	circle α
point B	**outside**	circle β

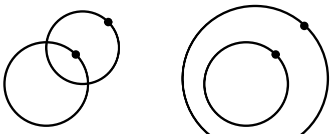

8

point A	**inside**	circle α
point A	**inside**	circle β
point B	**inside**	circle α
point B	**on**	circle β

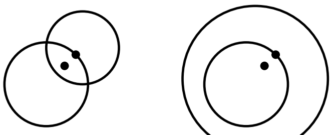

9

point A **on** circle α
point A **outside** circle β
point B **outside** circle α
point B **on** circle β

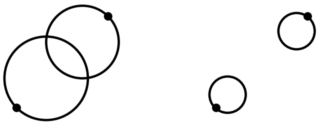

10

point A **inside** circle α
point A **outside** circle β
point B **outside** circle α
point B **on** circle β

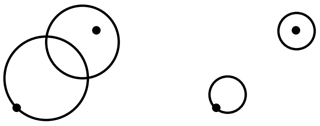

11

point A **inside** circle α
point A **outside** circle β
point B **on** circle α
point B **outside** circle β

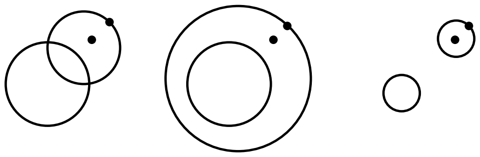

12

point A **inside** circle α
point A **inside** circle β
point B **inside** circle α
point B **inside** circle β

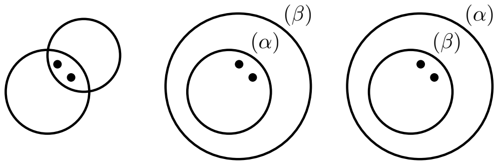

13

point A **inside** circle α
point A **inside** circle β
point B **outside** circle α
point B **outside** circle β

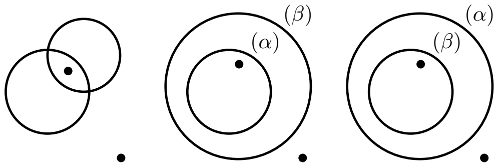

14

point A **inside** circle α
point A **outside** circle β
point B **inside** circle α
point B **outside** circle β

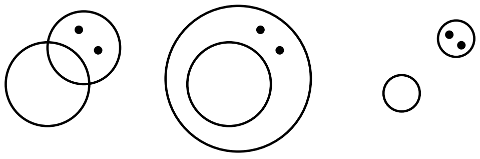

15

point A **inside** circle α
point A **outside** circle β
point B **outside** circle α
point B **outside** circle β

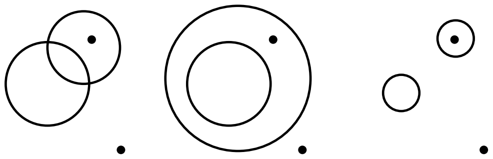

16

point A **outside** circle α
point A **outside** circle β
point B **outside** circle α
point B **outside** circle β

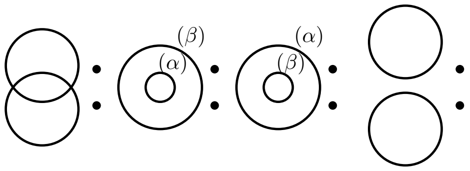

17

point A **on** circle α
point A **outside** circle β
point B **on** circle α
point B **outside** circle β

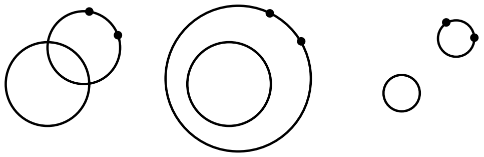

18

point A **on** circle α
point A **outside** circle β
point B **outside** circle α
point B **outside** circle β

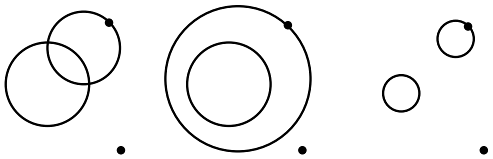

Figure S5. A screenshot of the reasoning task for Experiment 4. The setting was similar to that of Experiments 1 and 2, except for the presence of a possible representation of the premisses provided for each inference.

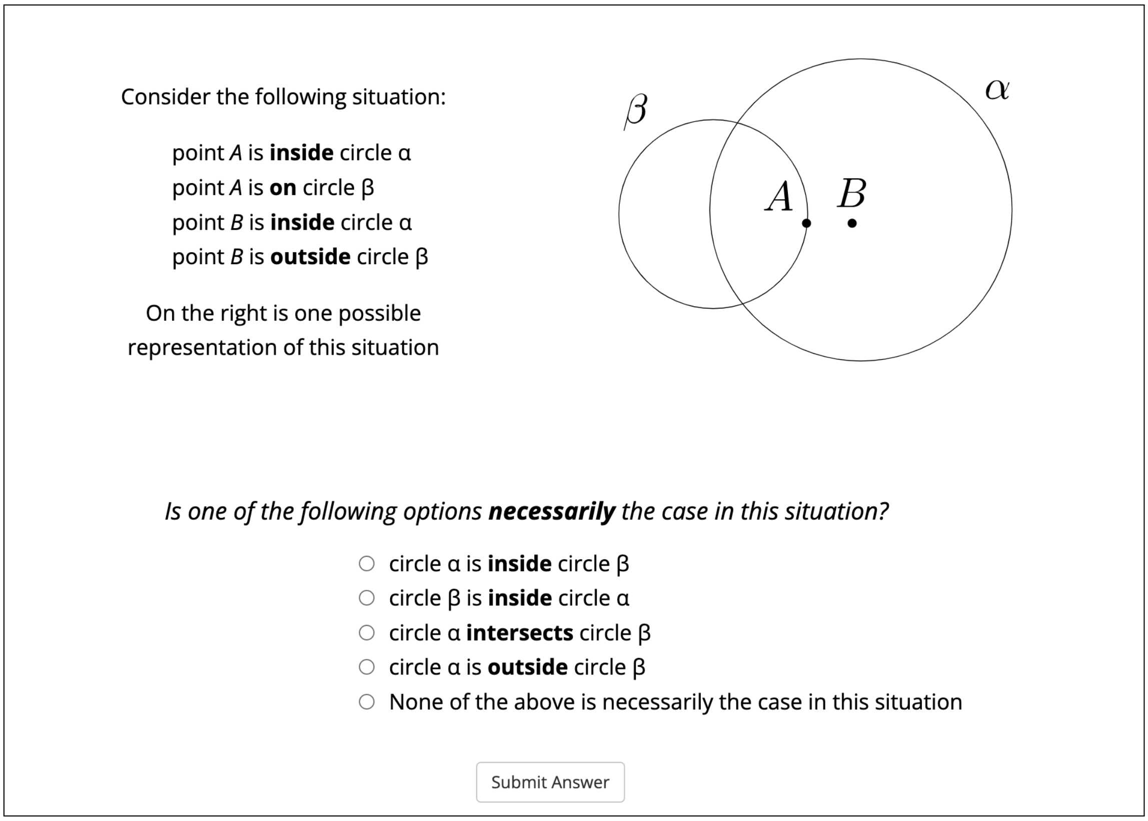

Figure S6. The 16 inferences tested in Experiment 4 together with the diagrams provided in each condition. From the set of inferences tested in Experiment 2, we picked 4 invalid inferences with the locking property, 4 invalid inferences without the locking property, and 8 valid inferences. Participants were divided in two groups. Participants assigned to the locked condition (group 1) saw the inferences together with the diagrams of the left column. Participants assigned to the non-locked condition (group 2) saw the inferences together with the diagrams of the right column. In the locked (resp. non-locked) condition, diagrams for invalid inferences with the locking property consisted of locked (resp. non-locked) configurations. The diagrams for invalid inferences without the locking property and for valid inferences were chosen to be as similar as possible to the diagrams provided for inferences with the locking property.

A. Invalid inferences with the locking property

1

point A	**inside**	circle α
point A	**inside**	circle β
point B	**inside**	circle α
point B	**outside**	circle β

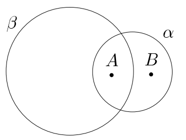

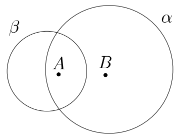

2

point A	**inside**	circle α
point A	**on**	circle β
point B	**inside**	circle α
point B	**outside**	circle β

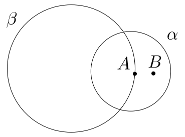

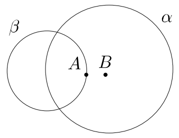

4

point A	**inside**	circle α
point A	**on**	circle β
point B	**inside**	circle α
point B	**on**	circle β

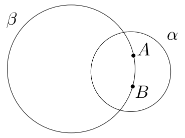

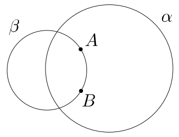

5

point A	**inside**	circle α
point A	**on**	circle β
point B	**outside**	circle α
point B	**outside**	circle β

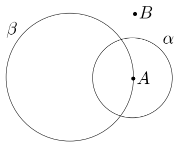

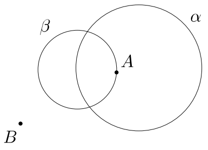

B. Invalid inferences without the locking property

**12**	point A **inside** circle α<br>point A **inside** circle β<br>point B **inside** circle α<br>point B **inside** circle β	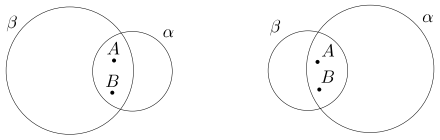
**13**	point A **inside** circle α<br>point A **inside** circle β<br>point B **outside** circle α<br>point B **outside** circle β	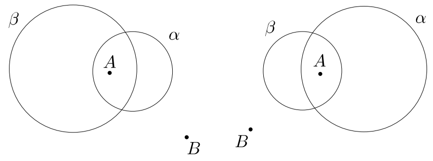
**14**	point A **inside** circle α<br>point A **outside** circle β<br>point B **inside** circle α<br>point B **outside** circle β	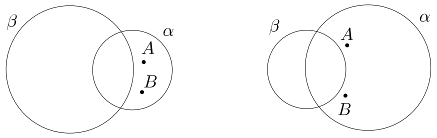
**17**	point A **on** circle α<br>point A **outside** circle β<br>point B **on** circle α<br>point B **outside** circle β	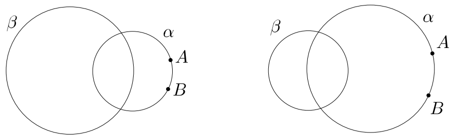

C. Valid inferences

1

point A **inside** circle α
point A **inside** circle β
point B **on** circle α
point B **on** circle β

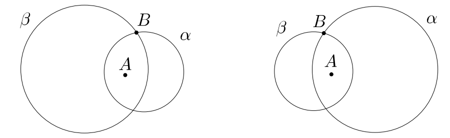

2

point A **inside** circle α
point A **on** circle β
point B **on** circle α
point B **inside** circle β

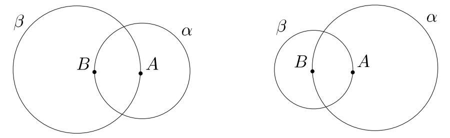

3

point A **inside** circle α
point A **on** circle β
point B **on** circle α
point B **on** circle β

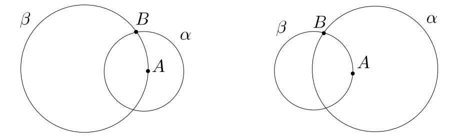

4

point A **inside** circle α
point A **on** circle β
point B **outside** circle α
point B **inside** circle β

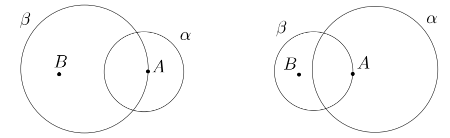

5

point A **inside** circle α
point A **on** circle β
point B **outside** circle α
point B **on** circle β

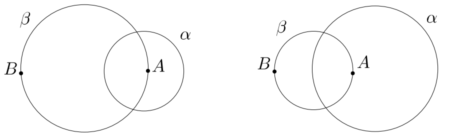

6

point A **inside** circle α
point A **outside** circle β
point B **on** circle α
point B **on** circle β

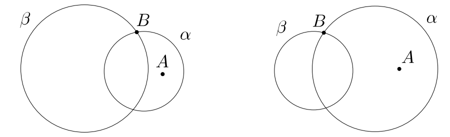

7

point A **on** circle α
point A **on** circle β
point B **on** circle α
point B **on** circle β

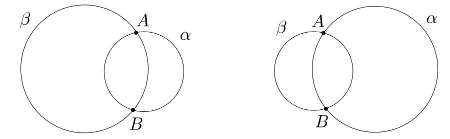

8

point A **on** circle α
point A **on** circle β
point B **on** circle α
point B **outside** circle β

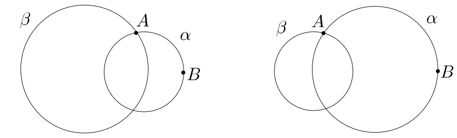

Table S1. The complete list of inferences tested in Experiments 1 and 2.

Experiment 1

Invalid Inferences

1, 2, 3, 4, 5, 6, 7, 8, 9, 10,
11, 12, 13, 14, 15, 16, 17, 18, 19, 20,
21, 22, 23, 24, 25, 26, 27, 28, 29, 30,
31, 32, 33, 34, 35, 36, 37, 38, 39, 40,
41, 42, 43, 44, 45, 46, 47, 48, 49, 50,
51, 52, 53, 54, 55, 56, 57, 58, 59, 60.

Valid Inferences

1, 2, 3, 4, 5, 6, 7, 8, 9, 10.

Experiment 2

Invalid Inferences

1, 2, 3, 4, 5, 6, 7, 8, 9, 10, 11, 12, 13, 14, 15, 16, 17, 18.

Valid Inferences

1, 2, 3, 4, 5, 6, 7, 8, 9, 10, 11, 12, 13, 14, 15, 16, 17, 18.

Experiment 1: List of All Invalid Inferences

Invalid inference 1 (Experiment 1)

Premisses:

point *A* is **inside** circle α
point *A* is **inside** circle β
point *B* is **inside** circle α
point *B* is **inside** circle β

Proposed Conclusions:

circle α is **inside** circle β
circle β is **inside** circle α
circle α **intersects** circle β
circle α is **outside** circle β
None of the above is necessarily the case in this situation

Correct Answer:

None of the above is necessarily the case in this situation

Invalid inference 2 (Experiment 1)

Premisses:

point *A* is **inside** circle α
point *A* is **inside** circle β
point *B* is **inside** circle α
point *B* is **on** circle β

Proposed Conclusions:

circle α is **inside** circle β
circle β is **inside** circle α
circle α **intersects** circle β
circle α is **outside** circle β
None of the above is necessarily the case in this situation

Correct Answer:

None of the above is necessarily the case in this situation

Invalid inference 3 (Experiment 1)

Premisses:

point *A* is **inside** circle α
point *A* is **inside** circle β
point *B* is **inside** circle α
point *B* is **outside** circle β

Proposed Conclusions:

circle α is **inside** circle β
circle β is **inside** circle α
circle α **intersects** circle β
circle α is **outside** circle β
None of the above is necessarily the case in this situation

Correct Answer:

None of the above is necessarily the case in this situation

Invalid inference 4 (Experiment 1)

Premisses:

point *A* is **inside** circle α
point *A* is **inside** circle β
point *B* is **on** circle α
point *B* is **outside** circle β

Proposed Conclusions:

circle α is **inside** circle β
circle β is **inside** circle α
circle α **intersects** circle β
circle α is **outside** circle β
None of the above is necessarily the case in this situation

Correct Answer:

None of the above is necessarily the case in this situation

Invalid inference 5 (Experiment 1)

Premisses:

point *A* is **inside** circle α
point *A* is **inside** circle β
point *B* is **outside** circle α
point *B* is **outside** circle β

Proposed Conclusions:

circle α is **inside** circle β
circle β is **inside** circle α
circle α **intersects** circle β
circle α is **outside** circle β
None of the above is necessarily the case in this situation

Correct Answer:

None of the above is necessarily the case in this situation

Invalid inference 6 (Experiment 1)

Premisses:

point *A* is **inside** circle α
point *A* is **on** circle β
point *B* is **inside** circle α
point *B* is **on** circle β

Proposed Conclusions:

circle α is **inside** circle β
circle β is **inside** circle α
circle α **intersects** circle β

circle α is **outside** circle β
None of the above is necessarily the case in this situation

Correct Answer:

None of the above is necessarily the case in this situation

Invalid inference 7 (Experiment 1)

Premisses:

point *A* is **inside** circle α
point *A* is **on** circle β
point *B* is **inside** circle α
point *B* is **outside** circle β

Proposed Conclusions:

circle α is **inside** circle β
circle β is **inside** circle α
circle α **intersects** circle β
circle α is **outside** circle β
None of the above is necessarily the case in this situation

Correct Answer:

None of the above is necessarily the case in this situation

Invalid inference 8 (Experiment 1)

Premisses:

point *A* is **inside** circle α
point *A* is **on** circle β
point *B* is **on** circle α
point *B* is **outside** circle β

Proposed Conclusions:

circle α is **inside** circle β
circle β is **inside** circle α
circle α **intersects** circle β
circle α is **outside** circle β
None of the above is necessarily the case in this situation

Correct Answer:

None of the above is necessarily the case in this situation

Invalid inference 9 (Experiment 1)

Premisses:

point *A* is **inside** circle α
point *A* is **on** circle β
point *B* is **outside** circle α
point *B* is **outside** circle β

Proposed Conclusions:

circle α is **inside** circle β
circle β is **inside** circle α
circle α **intersects** circle β
circle α is **outside** circle β
None of the above is necessarily the case in this situation

Correct Answer:

None of the above is necessarily the case in this situation

Invalid inference 10 (Experiment 1)

Premisses:

point *A* is **inside** circle α
point *A* is **outside** circle β
point *B* is **inside** circle α
point *B* is **outside** circle β

Proposed Conclusions:

circle α is **inside** circle β
circle β is **inside** circle α
circle α **intersects** circle β
circle α is **outside** circle β
None of the above is necessarily the case in this situation

Correct Answer:

None of the above is necessarily the case in this situation

Invalid inference 11 (Experiment 1)

Premisses:

point *A* is **inside** circle α
point *A* is **outside** circle β
point *B* is **on** circle α
point *B* is **outside** circle β

Proposed Conclusions:

circle α is **inside** circle β
circle β is **inside** circle α
circle α **intersects** circle β
circle α is **outside** circle β
None of the above is necessarily the case in this situation

Correct Answer:

None of the above is necessarily the case in this situation

Invalid inference 12 (Experiment 1)

Premisses:

point *A* is **inside** circle α
point *A* is **outside** circle β
point *B* is **outside** circle α
point *B* is **inside** circle β

Proposed Conclusions:

circle α is **inside** circle β
circle β is **inside** circle α
circle α **intersects** circle β
circle α is **outside** circle β
None of the above is necessarily the case in this situation

Correct Answer:

None of the above is necessarily the case in this situation

Invalid inference 13 (Experiment 1)

Premisses:

point *A* is **inside** circle α
point *A* is **outside** circle β
point *B* is **outside** circle α
point *B* is **on** circle β

Proposed Conclusions:

circle α is **inside** circle β
circle β is **inside** circle α
circle α **intersects** circle β
circle α is **outside** circle β
None of the above is necessarily the case in this situation

Correct Answer:

None of the above is necessarily the case in this situation

Invalid inference 14 (Experiment 1)

Premisses:

point *A* is **inside** circle α
point *A* is **outside** circle β
point *B* is **outside** circle α
point *B* is **outside** circle β

Proposed Conclusions:

circle α is **inside** circle β
circle β is **inside** circle α
circle α **intersects** circle β
circle α is **outside** circle β
None of the above is necessarily the case in this situation

Correct Answer:

None of the above is necessarily the case in this situation

Invalid inference 15 (Experiment 1)

Premisses:

point *A* is **on** circle α
point *A* is **outside** circle β
point *B* is **on** circle α
point *B* is **outside** circle β

Proposed Conclusions:

circle α is **inside** circle β
circle β is **inside** circle α
circle α **intersects** circle β
circle α is **outside** circle β
None of the above is necessarily the case in this situation

Correct Answer:

None of the above is necessarily the case in this situation

Invalid inference 16 (Experiment 1)

Premisses:

point *A* is **on** circle α
point *A* is **outside** circle β
point *B* is **outside** circle α
point *B* is **on** circle β

Proposed Conclusions:

circle α is **inside** circle β
circle β is **inside** circle α
circle α **intersects** circle β
circle α is **outside** circle β
None of the above is necessarily the case in this situation

Correct Answer:

None of the above is necessarily the case in this situation

Invalid inference 17 (Experiment 1)

Premisses:

point *A* is **on** circle α
point *A* is **outside** circle β
point *B* is **outside** circle α
point *B* is **outside** circle β

Proposed Conclusions:

circle α is **inside** circle β
circle β is **inside** circle α
circle α **intersects** circle β
circle α is **outside** circle β
None of the above is necessarily the case in this situation

Correct Answer:

None of the above is necessarily the case in this situation

Invalid inference 18 (Experiment 1)

Premisses:

point *A* is **outside** circle α
point *A* is **outside** circle β
point *B* is **outside** circle α
point *B* is **outside** circle β

Proposed Conclusions:

circle α is **inside** circle β
circle β is **inside** circle α
circle α **intersects** circle β
circle α is **outside** circle β
None of the above is necessarily the case in this situation

Correct Answer:

None of the above is necessarily the case in this situation

Invalid inference 19 (Experiment 1)

Premisses:

point *A* is **inside** circle α
point *A* is **inside** circle β
point *B* is **inside** circle α
circle β is **inside** circle α

Proposed Conclusions:

point *B* is **inside** circle β
point *B* is **on** circle β
point *B* is **outside** circle β
None of the above is necessarily the case in this situation

Correct Answer:

None of the above is necessarily the case in this situation

Invalid inference 20 (Experiment 1)

Premisses:

point *A* is **inside** circle α
point *A* is **inside** circle β
point *B* is **inside** circle α
circle α **intersects** circle β

Proposed Conclusions:

point *B* is **inside** circle β
point *B* is **on** circle β
point *B* is **outside** circle β
None of the above is necessarily the case in this situation

Correct Answer:

None of the above is necessarily the case in this situation

Invalid inference 21 (Experiment 1)

Premisses:

point *A* is **inside** circle α
point *A* is **inside** circle β
point *B* is **on** circle α
circle α **intersects** circle β

Proposed Conclusions:

point *B* is **inside** circle β
point *B* is **on** circle β
point *B* is **outside** circle β
None of the above is necessarily the case in this situation

Correct Answer:

None of the above is necessarily the case in this situation

Invalid inference 22 (Experiment 1)

Premisses:

point *A* is **inside** circle α
point *A* is **inside** circle β
point *B* is **outside** circle α
circle α is **inside** circle β

Proposed Conclusions:

point *B* is **inside** circle β
point *B* is **on** circle β
point *B* is **outside** circle β
None of the above is necessarily the case in this situation

Correct Answer:

None of the above is necessarily the case in this situation

Invalid inference 23 (Experiment 1)

Premisses:

point *A* is **inside** circle α
point *A* is **inside** circle β
point *B* is **outside** circle α
circle α **intersects** circle β

Proposed Conclusions:

point *B* is **inside** circle β
point *B* is **on** circle β
point *B* is **outside** circle β
None of the above is necessarily the case in this situation

Correct Answer:

None of the above is necessarily the case in this situation

Invalid inference 24 (Experiment 1)

Premisses:

point *A* is **inside** circle α
point *A* is **on** circle β
point *B* is **inside** circle α
circle β is **inside** circle α

Proposed Conclusions:

point *B* is **inside** circle β
point *B* is **on** circle β
point *B* is **outside** circle β
None of the above is necessarily the case in this situation

Correct Answer:

None of the above is necessarily the case in this situation

Invalid inference 25 (Experiment 1)

Premisses:

point *A* is **inside** circle α
point *A* is **on** circle β
point *B* is **inside** circle α
circle α **intersects** circle β

Proposed Conclusions:

point *B* is **inside** circle β
point *B* is **on** circle β
point *B* is **outside** circle β
None of the above is necessarily the case in this situation

Correct Answer:

None of the above is necessarily the case in this situation

Invalid inference 26 (Experiment 1)

Premisses:

point *A* is **inside** circle α
point *A* is **on** circle β
point *B* is **on** circle α
circle α **intersects** circle β

Proposed Conclusions:

point *B* is **inside** circle β
point *B* is **on** circle β
point *B* is **outside** circle β
None of the above is necessarily the case in this situation

Correct Answer:

None of the above is necessarily the case in this situation

Invalid inference 27 (Experiment 1)

Premisses:

point *A* is **inside** circle α
point *A* is **on** circle β
point *B* is **outside** circle α
circle α **intersects** circle β

Proposed Conclusions:

point *B* is **inside** circle β
point *B* is **on** circle β
point *B* is **outside** circle β
None of the above is necessarily the case in this situation

Correct Answer:

None of the above is necessarily the case in this situation

Invalid inference 28 (Experiment 1)

Premisses:

point *A* is **inside** circle α
point *A* is **outside** circle β
point *B* is **inside** circle α
circle β is **inside** circle α

Proposed Conclusions:

point *B* is **inside** circle β
point *B* is **on** circle β
point *B* is **outside** circle β
None of the above is necessarily the case in this situation

Correct Answer:

None of the above is necessarily the case in this situation

Invalid inference 29 (Experiment 1)

Premisses:

point *A* is **inside** circle α
point *A* is **outside** circle β
point *B* is **inside** circle α
circle α **intersects** circle β

Proposed Conclusions:

point *B* is **inside** circle β
point *B* is **on** circle β
point *B* is **outside** circle β
None of the above is necessarily the case in this situation

Correct Answer:

None of the above is necessarily the case in this situation

Invalid inference 30 (Experiment 1)

Premisses:

point *A* is **inside** circle α
point *A* is **outside** circle β
point *B* is **on** circle α
circle α **intersects** circle β

Proposed Conclusions:

point *B* is **inside** circle β
point *B* is **on** circle β
point *B* is **outside** circle β
None of the above is necessarily the case in this situation

Correct Answer:

None of the above is necessarily the case in this situation

Invalid inference 31 (Experiment 1)

Premisses:

point *A* is **inside** circle α
point *A* is **outside** circle β
point *B* is **outside** circle α
circle α **intersects** circle β

Proposed Conclusions:

point *B* is **inside** circle β
point *B* is **on** circle β

point *B* is **outside** circle β
None of the above is necessarily the case in this situation

Correct Answer:

None of the above is necessarily the case in this situation

Invalid inference 32 (Experiment 1)

Premisses:

point *A* is **inside** circle α
point *A* is **outside** circle β
point *B* is **outside** circle α
circle α is **outside** circle β

Proposed Conclusions:

point *B* is **inside** circle β
point *B* is **on** circle β
point *B* is **outside** circle β
None of the above is necessarily the case in this situation

Correct Answer:

None of the above is necessarily the case in this situation

Invalid inference 33 (Experiment 1)

Premisses:

point *A* is **on** circle α
point *A* is **inside** circle β
point *B* is **inside** circle α
circle α **intersects** circle β

Proposed Conclusions:

point *B* is **inside** circle β
point *B* is **on** circle β
point *B* is **outside** circle β
None of the above is necessarily the case in this situation

Correct Answer:

None of the above is necessarily the case in this situation

Invalid inference 34 (Experiment 1)

Premisses:

point *A* is **on** circle α
point *A* is **inside** circle β
point *B* is **on** circle α
circle α **intersects** circle β

Proposed Conclusions:

point *B* is **inside** circle β
point *B* is **on** circle β
point *B* is **outside** circle β
None of the above is necessarily the case in this situation

Correct Answer:

None of the above is necessarily the case in this situation

Invalid inference 35 (Experiment 1)

Premisses:

point *A* is **on** circle α
point *A* is **inside** circle β
point *B* is **outside** circle α
circle α is **inside** circle β

Proposed Conclusions:

point *B* is **inside** circle β
point *B* is **on** circle β
point *B* is **outside** circle β
None of the above is necessarily the case in this situation

Correct Answer:

None of the above is necessarily the case in this situation

Invalid inference 36 (Experiment 1)

Premisses:

point *A* is **on** circle α
point *A* is **inside** circle β
point *B* is **outside** circle α
circle α **intersects** circle β

Proposed Conclusions:

point *B* is **inside** circle β
point *B* is **on** circle β
point *B* is **outside** circle β
None of the above is necessarily the case in this situation

Correct Answer:

None of the above is necessarily the case in this situation

Invalid inference 37 (Experiment 1)

Premisses:

point *A* is **on** circle α
point *A* is **on** circle β
point *B* is **inside** circle α
circle α **intersects** circle β

Proposed Conclusions:

point *B* is **inside** circle β
point *B* is **on** circle β
point *B* is **outside** circle β
None of the above is necessarily the case in this situation

Correct Answer:

None of the above is necessarily the case in this situation

Invalid inference 38 (Experiment 1)

Premisses:

point *A* is **on** circle α
point *A* is **on** circle β
point *B* is **on** circle α
circle α **intersects** circle β

Proposed Conclusions:

point *B* is **inside** circle β
point *B* is **on** circle β
point *B* is **outside** circle β
None of the above is necessarily the case in this situation

Correct Answer:

None of the above is necessarily the case in this situation

Invalid inference 39 (Experiment 1)

Premisses:

point *A* is **on** circle α
point *A* is **on** circle β

point *B* is **outside** circle α
circle α **intersects** circle β

Proposed Conclusions:

point *B* is **inside** circle β
point *B* is **on** circle β
point *B* is **outside** circle β
None of the above is necessarily the case in this situation

Correct Answer:

None of the above is necessarily the case in this situation

Invalid inference 40 (Experiment 1)

Premisses:

point *A* is **on** circle α
point *A* is **outside** circle β
point *B* is **inside** circle α
circle β is **inside** circle α

Proposed Conclusions:

point *B* is **inside** circle β
point *B* is **on** circle β
point *B* is **outside** circle β
None of the above is necessarily the case in this situation

Correct Answer:

None of the above is necessarily the case in this situation

Invalid inference 41 (Experiment 1)

Premisses:

point *A* is **on** circle α
point *A* is **outside** circle β
point *B* is **inside** circle α
circle α **intersects** circle β

Proposed Conclusions:

point *B* is **inside** circle β
point *B* is **on** circle β
point *B* is **outside** circle β
None of the above is necessarily the case in this situation

Correct Answer:

None of the above is necessarily the case in this situation

Invalid inference 42 (Experiment 1)

Premisses:

point *A* is **on** circle α
point *A* is **outside** circle β
point *B* is **on** circle α
circle α **intersects** circle β

Proposed Conclusions:

point *B* is **inside** circle β
point *B* is **on** circle β
point *B* is **outside** circle β
None of the above is necessarily the case in this situation

Correct Answer:

None of the above is necessarily the case in this situation

Invalid inference 43 (Experiment 1)

Premisses:

point *A* is **on** circle α
point *A* is **outside** circle β
point *B* is **outside** circle α
circle α **intersects** circle β

Proposed Conclusions:

point *B* is **inside** circle β
point *B* is **on** circle β
point *B* is **outside** circle β
None of the above is necessarily the case in this situation

Correct Answer:

None of the above is necessarily the case in this situation

Invalid inference 44 (Experiment 1)

Premisses:

point *A* is **on** circle α
point *A* is **outside** circle β
point *B* is **outside** circle α
circle α is **outside** circle β

Proposed Conclusions:

point *B* is **inside** circle β
point *B* is **on** circle β
point *B* is **outside** circle β
None of the above is necessarily the case in this situation

Correct Answer:

None of the above is necessarily the case in this situation

Invalid inference 45 (Experiment 1)

Premisses:

point *A* is **outside** circle α
point *A* is **inside** circle β
point *B* is **inside** circle α
circle α **intersects** circle β

Proposed Conclusions:

point *B* is **inside** circle β
point *B* is **on** circle β
point *B* is **outside** circle β
None of the above is necessarily the case in this situation

Correct Answer:

None of the above is necessarily the case in this situation

Invalid inference 46 (Experiment 1)

Premisses:

point *A* is **outside** circle α
point *A* is **inside** circle β
point *B* is **on** circle α
circle α **intersects** circle β

Proposed Conclusions:

point *B* is **inside** circle β
point *B* is **on** circle β
point *B* is **outside** circle β
None of the above is necessarily the case in this situation

Correct Answer:

None of the above is necessarily the case in this situation

Invalid inference 47 (Experiment 1)

Premisses:

point *A* is **outside** circle α
point *A* is **inside** circle β
point *B* is **outside** circle α
circle α is **inside** circle β

Proposed Conclusions:

point *B* is **inside** circle β
point *B* is **on** circle β
point *B* is **outside** circle β
None of the above is necessarily the case in this situation

Correct Answer:

None of the above is necessarily the case in this situation

Invalid inference 48 (Experiment 1)

Premisses:

point *A* is **outside** circle α
point *A* is **inside** circle β
point *B* is **outside** circle α
circle α **intersects** circle β

Proposed Conclusions:

point *B* is **inside** circle β
point *B* is **on** circle β
point *B* is **outside** circle β
None of the above is necessarily the case in this situation

Correct Answer:

None of the above is necessarily the case in this situation

Invalid inference 49 (Experiment 1)

Premisses:

point *A* is **outside** circle α
point *A* is **inside** circle β
point *B* is **outside** circle α
circle α is **outside** circle β

Proposed Conclusions:

point *B* is **inside** circle β
point *B* is **on** circle β
point *B* is **outside** circle β
None of the above is necessarily the case in this situation

Correct Answer:

None of the above is necessarily the case in this situation

Invalid inference 50 (Experiment 1)

Premisses:

point *A* is **outside** circle α
point *A* is **on** circle β
point *B* is **inside** circle α
circle α **intersects** circle β

Proposed Conclusions:

point *B* is **inside** circle β
point *B* is **on** circle β
point *B* is **outside** circle β
None of the above is necessarily the case in this situation

Correct Answer:

None of the above is necessarily the case in this situation

Invalid inference 51 (Experiment 1)

Premisses:

point *A* is **outside** circle α
point *A* is **on** circle β
point *B* is **on** circle α
circle α **intersects** circle β

Proposed Conclusions:

point *B* is **inside** circle β
point *B* is **on** circle β
point *B* is **outside** circle β
None of the above is necessarily the case in this situation

Correct Answer:

None of the above is necessarily the case in this situation

Invalid inference 52 (Experiment 1)

Premisses:

point *A* is **outside** circle α
point *A* is **on** circle β
point *B* is **outside** circle α
circle α is **inside** circle β

Proposed Conclusions:

point *B* is **inside** circle β
point *B* is **on** circle β
point *B* is **outside** circle β
None of the above is necessarily the case in this situation

Correct Answer:

None of the above is necessarily the case in this situation

Invalid inference 53 (Experiment 1)

Premisses:

point *A* is **outside** circle α
point *A* is **on** circle β
point *B* is **outside** circle α
circle α **intersects** circle β

Proposed Conclusions:

point *B* is **inside** circle β
point *B* is **on** circle β
point *B* is **outside** circle β
None of the above is necessarily the case in this situation

Correct Answer:

None of the above is necessarily the case in this situation

Invalid inference 54 (Experiment 1)

Premisses:

point *A* is **outside** circle α
point *A* is **on** circle β
point *B* is **outside** circle α
circle α is **outside** circle β

Proposed Conclusions:

point *B* is **inside** circle β
point *B* is **on** circle β

point *B* is **outside** circle β
None of the above is necessarily the case in this situation

Correct Answer:

None of the above is necessarily the case in this situation

Invalid inference 55 (Experiment 1)

Premisses:

point *A* is **outside** circle α
point *A* is **outside** circle β
point *B* is **inside** circle α
circle β is **inside** circle α

Proposed Conclusions:

point *B* is **inside** circle β
point *B* is **on** circle β
point *B* is **outside** circle β
None of the above is necessarily the case in this situation

Correct Answer:

None of the above is necessarily the case in this situation

Invalid inference 56 (Experiment 1)

Premisses:

point *A* is **outside** circle α
point *A* is **outside** circle β
point *B* is **inside** circle α
circle α **intersects** circle β

Proposed Conclusions:

point *B* is **inside** circle β
point *B* is **on** circle β
point *B* is **outside** circle β
None of the above is necessarily the case in this situation

Correct Answer:

None of the above is necessarily the case in this situation

Invalid inference 57 (Experiment 1)

Premisses:

point *A* is **outside** circle α
point *A* is **outside** circle β
point *B* is **on** circle α
circle α **intersects** circle β

Proposed Conclusions:

point *B* is **inside** circle β
point *B* is **on** circle β
point *B* is **outside** circle β
None of the above is necessarily the case in this situation

Correct Answer:

None of the above is necessarily the case in this situation

Invalid inference 58 (Experiment 1)

Premisses:

point *A* is **outside** circle α
point *A* is **outside** circle β
point *B* is **outside** circle α
circle α is **inside** circle β

Proposed Conclusions:

point *B* is **inside** circle β
point *B* is **on** circle β
point *B* is **outside** circle β
None of the above is necessarily the case in this situation

Correct Answer:

None of the above is necessarily the case in this situation

Invalid inference 59 (Experiment 1)

Premisses:

point *A* is **outside** circle α
point *A* is **outside** circle β
point *B* is **outside** circle α
circle α **intersects** circle β

Proposed Conclusions:

point *B* is **inside** circle β
point *B* is **on** circle β
point *B* is **outside** circle β
None of the above is necessarily the case in this situation

Correct Answer:

None of the above is necessarily the case in this situation

Invalid inference 60 (Experiment 1)

Premisses:

point *A* is **outside** circle α
point *A* is **outside** circle β
point *B* is **outside** circle α
circle α is **outside** circle β

Proposed Conclusions:

point *B* is **inside** circle β
point *B* is **on** circle β
point *B* is **outside** circle β
None of the above is necessarily the case in this situation

Correct Answer:

None of the above is necessarily the case in this situation

Experiment 1: List of All Valid Inferences

Valid inference 1 (Experiment 1)

Premisses:

point *A* is **inside** circle α
point *A* is **on** circle β
point *B* is **on** circle α
point *B* is **inside** circle β

Proposed Conclusions:

circle α is **inside** circle β
circle β is **inside** circle α
circle α **intersects** circle β
circle α is **outside** circle β
None of the above is necessarily the case in this situation

Correct Answer:

circle α **intersects** circle β

Valid inference 2 (Experiment 1)

Premisses:

point *A* is **inside** circle α
point *A* is **on** circle β
point *B* is **outside** circle α
point *B* is **inside** circle β

Proposed Conclusions:

circle α is **inside** circle β
circle β is **inside** circle α
circle α **intersects** circle β
circle α is **outside** circle β
None of the above is necessarily the case in this situation

Correct Answer:

circle α **intersects** circle β

Valid inference 3 (Experiment 1)

Premisses:

point *A* is **on** circle α
point *A* is **on** circle β
point *B* is **on** circle α
point *B* is **on** circle β

Proposed Conclusions:

circle α is **inside** circle β
circle β is **inside** circle α
circle α **intersects** circle β
circle α is **outside** circle β
None of the above is necessarily the case in this situation

Correct Answer:

circle α **intersects** circle β

Valid inference 4 (Experiment 1)

Premisses:

point *A* is **inside** circle α
point *A* is **on** circle β
point *B* is **outside** circle α
point *B* is **on** circle β

Proposed Conclusions:

circle α is **inside** circle β
circle β is **inside** circle α
circle α **intersects** circle β

circle α is **outside** circle β
None of the above is necessarily the case in this situation

Correct Answer:

circle α **intersects** circle β

Valid inference 5 (Experiment 1)

Premisses:

point *A* is **outside** circle α
point *A* is **outside** circle β
point *B* is **outside** circle α
circle β is **inside** circle α

Proposed Conclusions:

point *B* is **inside** circle β
point *B* is **on** circle β
point *B* is **outside** circle β
None of the above is necessarily the case in this situation

Correct Answer:

point *B* is **outside** circle β

Valid inference 6 (Experiment 1)

Premisses:

point *A* is **outside** circle α
point *A* is **outside** circle β
point *B* is **on** circle α
circle α is **outside** circle β

Proposed Conclusions:

point *B* is **inside** circle β
point *B* is **on** circle β
point *B* is **outside** circle β
None of the above is necessarily the case in this situation

Correct Answer:

point *B* is **outside** circle β

Valid inference 7 (Experiment 1)

Premisses:

point *A* is **outside** circle α
point *A* is **outside** circle β
point *B* is **on** circle α
circle α is **inside** circle β

Proposed Conclusions:

point *B* is **inside** circle β
point *B* is **on** circle β
point *B* is **outside** circle β
None of the above is necessarily the case in this situation

Correct Answer:

point *B* is **inside** circle β

Valid inference 8 (Experiment 1)

Premisses:

point *A* is **outside** circle α
point *A* is **outside** circle β
point *B* is **inside** circle α
circle α is **outside** circle β

Proposed Conclusions:

point *B* is **inside** circle β
point *B* is **on** circle β
point *B* is **outside** circle β
None of the above is necessarily the case in this situation

Correct Answer:

point *B* is **outside** circle β

Valid inference 9 (Experiment 1)

Premisses:

point *A* is **outside** circle α
point *A* is **outside** circle β
point *B* is **inside** circle α
circle α is **inside** circle β

Proposed Conclusions:

point *B* is **inside** circle β
point *B* is **on** circle β
point *B* is **outside** circle β
None of the above is necessarily the case in this situation

Correct Answer:

point *B* is **inside** circle β

Valid inference 10 (Experiment 1)

Premisses:

point *A* is **inside** circle α
point *A* is **inside** circle β
point *B* is **inside** circle α
circle α is **inside** circle β

Proposed Conclusions:

point *B* is **inside** circle β
point *B* is **on** circle β
point *B* is **outside** circle β
None of the above is necessarily the case in this situation

Correct Answer:

point *B* is **inside** circle β

Experiment 2: List of All Invalid Inferences

Invalid inference 1 (Experiment 2)

Premisses:

point *A* is **inside** circle α
point *A* is **inside** circle β
point *B* is **inside** circle α
point *B* is **outside** circle β

Proposed Conclusions:

circle α is **inside** circle β
circle β is **inside** circle α
circle α **intersects** circle β
circle α is **outside** circle β
None of the above is necessarily the case in this situation

Correct Answer:

None of the above is necessarily the case in this situation

Invalid inference 2 (Experiment 2)

Premisses:

point *A* is **inside** circle α
point *A* is **on** circle β
point *B* is **inside** circle α
point *B* is **outside** circle β

Proposed Conclusions:

circle α is **inside** circle β
circle β is **inside** circle α
circle α **intersects** circle β
circle α is **outside** circle β
None of the above is necessarily the case in this situation

Correct Answer:

None of the above is necessarily the case in this situation

Invalid inference 3 (Experiment 2)

Premisses:

point *A* is **inside** circle α
point *A* is **inside** circle β
point *B* is **on** circle α
point *B* is **outside** circle β

Proposed Conclusions:

circle α is **inside** circle β
circle β is **inside** circle α
circle α **intersects** circle β
circle α is **outside** circle β
None of the above is necessarily the case in this situation

Correct Answer:

None of the above is necessarily the case in this situation

Invalid inference 4 (Experiment 2)

Premisses:

point *A* is **inside** circle α
point *A* is **on** circle β
point *B* is **inside** circle α
point *B* is **on** circle β

Proposed Conclusions:

circle α is **inside** circle β
circle β is **inside** circle α
circle α **intersects** circle β

circle α is **outside** circle β
None of the above is necessarily the case in this situation

Correct Answer:

None of the above is necessarily the case in this situation

Invalid inference 5 (Experiment 2)

Premisses:

point *A* is **inside** circle α
point *A* is **on** circle β
point *B* is **outside** circle α
point *B* is **outside** circle β

Proposed Conclusions:

circle α is **inside** circle β
circle β is **inside** circle α
circle α **intersects** circle β
circle α is **outside** circle β
None of the above is necessarily the case in this situation

Correct Answer:

None of the above is necessarily the case in this situation

Invalid inference 6 (Experiment 2)

Premisses:

point *A* is **inside** circle α
point *A* is **outside** circle β
point *B* is **outside** circle α
point *B* is **inside** circle β

Proposed Conclusions:

circle α is **inside** circle β
circle β is **inside** circle α
circle α **intersects** circle β
circle α is **outside** circle β
None of the above is necessarily the case in this situation

Correct Answer:

None of the above is necessarily the case in this situation

Invalid inference 7 (Experiment 2)

Premisses:

point *A* is **inside** circle α
point *A* is **on** circle β
point *B* is **on** circle α
point *B* is **outside** circle β

Proposed Conclusions:

circle α is **inside** circle β
circle β is **inside** circle α
circle α **intersects** circle β
circle α is **outside** circle β
None of the above is necessarily the case in this situation

Correct Answer:

None of the above is necessarily the case in this situation

Invalid inference 8 (Experiment 2)

Premisses:

point *A* is **inside** circle α
point *A* is **inside** circle β
point *B* is **inside** circle α
point *B* is **on** circle β

Proposed Conclusions:

circle α is **inside** circle β
circle β is **inside** circle α
circle α **intersects** circle β
circle α is **outside** circle β
None of the above is necessarily the case in this situation

Correct Answer:

None of the above is necessarily the case in this situation

Invalid inference 9 (Experiment 2)

Premisses:

point *A* is **on** circle α
point *A* is **outside** circle β
point *B* is **outside** circle α
point *B* is **on** circle β

Proposed Conclusions:

circle α is **inside** circle β
circle β is **inside** circle α
circle α **intersects** circle β
circle α is **outside** circle β
None of the above is necessarily the case in this situation

Correct Answer:

None of the above is necessarily the case in this situation

Invalid inference 10 (Experiment 2)

Premisses:

point *A* is **inside** circle α
point *A* is **outside** circle β
point *B* is **outside** circle α
point *B* is **on** circle β

Proposed Conclusions:

circle α is **inside** circle β
circle β is **inside** circle α
circle α **intersects** circle β
circle α is **outside** circle β
None of the above is necessarily the case in this situation

Correct Answer:

None of the above is necessarily the case in this situation

Invalid inference 11 (Experiment 2)

Premisses:

point *A* is **inside** circle α
point *A* is **outside** circle β
point *B* is **on** circle α
point *B* is **outside** circle β

Proposed Conclusions:

circle α is **inside** circle β
circle β is **inside** circle α
circle α **intersects** circle β
circle α is **outside** circle β
None of the above is necessarily the case in this situation

Correct Answer:

None of the above is necessarily the case in this situation

Invalid inference 12 (Experiment 2)

Premisses:

point *A* is **inside** circle α
point *A* is **inside** circle β
point *B* is **inside** circle α
point *B* is **inside** circle β

Proposed Conclusions:

circle α is **inside** circle β
circle β is **inside** circle α
circle α **intersects** circle β
circle α is **outside** circle β
None of the above is necessarily the case in this situation

Correct Answer:

None of the above is necessarily the case in this situation

Invalid inference 13 (Experiment 2)

Premisses:

point *A* is **inside** circle α
point *A* is **inside** circle β
point *B* is **outside** circle α
point *B* is **outside** circle β

Proposed Conclusions:

circle α is **inside** circle β
circle β is **inside** circle α
circle α **intersects** circle β
circle α is **outside** circle β
None of the above is necessarily the case in this situation

Correct Answer:

None of the above is necessarily the case in this situation

Invalid inference 14 (Experiment 2)

Premisses:

point *A* is **inside** circle α
point *A* is **outside** circle β
point *B* is **inside** circle α
point *B* is **outside** circle β

Proposed Conclusions:

circle α is **inside** circle β
circle β is **inside** circle α
circle α **intersects** circle β
circle α is **outside** circle β
None of the above is necessarily the case in this situation

Correct Answer:

None of the above is necessarily the case in this situation

Invalid inference 15 (Experiment 2)

Premisses:

point *A* is **inside** circle α
point *A* is **outside** circle β
point *B* is **outside** circle α
point *B* is **outside** circle β

Proposed Conclusions:

circle α is **inside** circle β
circle β is **inside** circle α
circle α **intersects** circle β
circle α is **outside** circle β
None of the above is necessarily the case in this situation

Correct Answer:

None of the above is necessarily the case in this situation

Invalid inference 16 (Experiment 2)

Premisses:

point *A* is **outside** circle α
point *A* is **outside** circle β
point *B* is **outside** circle α
point *B* is **outside** circle β

Proposed Conclusions:

circle α is **inside** circle β
circle β is **inside** circle α
circle α **intersects** circle β
circle α is **outside** circle β
None of the above is necessarily the case in this situation

Correct Answer:

None of the above is necessarily the case in this situation

Invalid inference 17 (Experiment 2)

Premisses:

point *A* is **on** circle α
point *A* is **outside** circle β
point *B* is **on** circle α
point *B* is **outside** circle β

Proposed Conclusions:

circle α is **inside** circle β
circle β is **inside** circle α
circle α **intersects** circle β
circle α is **outside** circle β
None of the above is necessarily the case in this situation

Correct Answer:

None of the above is necessarily the case in this situation

Invalid inference 18 (Experiment 2)

Premisses:

point *A* is **on** circle α
point *A* is **outside** circle β
point *B* is **outside** circle α
point *B* is **outside** circle β

Proposed Conclusions:

circle α is **inside** circle β
circle β is **inside** circle α
circle α **intersects** circle β
circle α is **outside** circle β
None of the above is necessarily the case in this situation

Correct Answer:

None of the above is necessarily the case in this situation

Experiment 2: List of All Valid Inferences

Valid inference 1 (Experiment 2)

Premisses:

point *A* is **inside** circle α
point *A* is **inside** circle β
point *B* is **on** circle α
point *B* is **on** circle β

Proposed Conclusions:

circle α is **inside** circle β
circle β is **inside** circle α
circle α **intersects** circle β
circle α is **outside** circle β
None of the above is necessarily the case in this situation

Correct Answer:

circle α **intersects** circle β

Valid inference 2 (Experiment 2)

Premisses:

point *A* is **inside** circle α
point *A* is **on** circle β
point *B* is **on** circle α
point *B* is **inside** circle β

Proposed Conclusions:

circle α is **inside** circle β
circle β is **inside** circle α
circle α **intersects** circle β
circle α is **outside** circle β
None of the above is necessarily the case in this situation

Correct Answer:

circle α **intersects** circle β

Valid inference 3 (Experiment 2)

Premisses:

point *A* is **inside** circle α
point *A* is **on** circle β
point *B* is **on** circle α
point *B* is **on** circle β

Proposed Conclusions:

circle α is **inside** circle β
circle β is **inside** circle α
circle α **intersects** circle β

circle α is **outside** circle β
None of the above is necessarily the case in this situation

Correct Answer:

circle α **intersects** circle β

Valid inference 4 (Experiment 2)

Premisses:

point *A* is **inside** circle α
point *A* is **on** circle β
point *B* is **outside** circle α
point *B* is **inside** circle β

Proposed Conclusions:

circle α is **inside** circle β
circle β is **inside** circle α
circle α **intersects** circle β
circle α is **outside** circle β
None of the above is necessarily the case in this situation

Correct Answer:

circle α **intersects** circle β

Valid inference 5 (Experiment 2)

Premisses:

point *A* is **inside** circle α
point *A* is **on** circle β
point *B* is **outside** circle α
point *B* is **on** circle β

Proposed Conclusions:

circle α is **inside** circle β
circle β is **inside** circle α
circle α **intersects** circle β
circle α is **outside** circle β
None of the above is necessarily the case in this situation

Correct Answer:

circle α **intersects** circle β

Valid inference 6 (Experiment 2)

Premisses:

point *A* is **inside** circle α
point *A* is **outside** circle β
point *B* is **on** circle α
point *B* is **on** circle β

Proposed Conclusions:

circle α is **inside** circle β
circle β is **inside** circle α
circle α **intersects** circle β
circle α is **outside** circle β
None of the above is necessarily the case in this situation

Correct Answer:

circle α **intersects** circle β

Valid inference 7 (Experiment 2)

Premisses:

point *A* is **on** circle α
point *A* is **on** circle β
point *B* is **on** circle α
point *B* is **on** circle β

Proposed Conclusions:

circle α is **inside** circle β
circle β is **inside** circle α
circle α **intersects** circle β
circle α is **outside** circle β
None of the above is necessarily the case in this situation

Correct Answer:

circle α **intersects** circle β

Valid inference 8 (Experiment 2)

Premisses:

point *A* is **on** circle α
point *A* is **on** circle β
point *B* is **on** circle α
point *B* is **outside** circle β

Proposed Conclusions:

circle α is **inside** circle β
circle β is **inside** circle α
circle α **intersects** circle β
circle α is **outside** circle β
None of the above is necessarily the case in this situation

Correct Answer:

circle α **intersects** circle β

Valid inference 9 (Experiment 2)

Premisses:

point *A* is **on** circle α
point *A* is **on** circle β
point *B* is **outside** circle α
point *B* is **outside** circle β

Proposed Conclusions:

circle α is **inside** circle β
circle β is **inside** circle α
circle α **intersects** circle β
circle α is **outside** circle β
None of the above is necessarily the case in this situation

Correct Answer:

circle α **intersects** circle β

Valid inference 10 (Experiment 2)

Premisses:

point *A* is **inside** circle α
point *A* is **inside** circle β
point *B* is **inside** circle α
circle α is **inside** circle β

Proposed Conclusions:

point *B* is **inside** circle β
point *B* is **on** circle β
point *B* is **outside** circle β
None of the above is necessarily the case in this situation

Correct Answer:

point *B* is **inside** circle β

Valid inference 11 (Experiment 2)

Premisses:

point *A* is **inside** circle α
point *A* is **inside** circle β
point *B* is **on** circle α
circle α is **inside** circle β

Proposed Conclusions:

point *B* is **inside** circle β
point *B* is **on** circle β
point *B* is **outside** circle β
None of the above is necessarily the case in this situation

Correct Answer:

point *B* is **inside** circle β

Valid inference 12 (Experiment 2)

Premisses:

point *A* is **inside** circle α
point *A* is **inside** circle β
point *B* is **outside** circle α
circle β is **inside** circle α

Proposed Conclusions:

point *B* is **inside** circle β
point *B* is **on** circle β
point *B* is **outside** circle β
None of the above is necessarily the case in this situation

Correct Answer:

point *B* is **outside** circle β

Valid inference 13 (Experiment 2)

Premisses:

point *A* is **inside** circle α
point *A* is **on** circle β
point *B* is **on** circle α
circle β is **inside** circle α

Proposed Conclusions:

point *B* is **inside** circle β
point *B* is **on** circle β
point *B* is **outside** circle β
None of the above is necessarily the case in this situation

Correct Answer:

point *B* is **outside** circle β

Valid inference 14 (Experiment 2)

Premisses:

point *A* is **outside** circle α
point *A* is **outside** circle β
point *B* is **outside** circle α
circle β is **inside** circle α

Proposed Conclusions:

point *B* is **inside** circle β
point *B* is **on** circle β
point *B* is **outside** circle β
None of the above is necessarily the case in this situation

Correct Answer:

point *B* is **outside** circle β

Valid inference 15 (Experiment 2)

Premisses:

point *A* is **outside** circle α
point *A* is **outside** circle β
point *B* is **on** circle α
circle α is **outside** circle β

Proposed Conclusions:

point *B* is **inside** circle β
point *B* is **on** circle β
point *B* is **outside** circle β
None of the above is necessarily the case in this situation

Correct Answer:

point *B* is **outside** circle β

Valid inference 16 (Experiment 2)

Premisses:

point *A* is **outside** circle α
point *A* is **outside** circle β
point *B* is **on** circle α
circle α is **inside** circle β

Proposed Conclusions:

point *B* is **inside** circle β
point *B* is **on** circle β
point *B* is **outside** circle β
None of the above is necessarily the case in this situation

Correct Answer:

point *B* is **inside** circle β

Valid inference 17 (Experiment 2)

Premisses:

point *A* is **outside** circle α
point *A* is **outside** circle β
point *B* is **inside** circle α
circle α is **outside** circle β

Proposed Conclusions:

point *B* is **inside** circle β
point *B* is **on** circle β
point *B* is **outside** circle β
None of the above is necessarily the case in this situation

Correct Answer:

point *B* is **outside** circle β

Valid inference 18 (Experiment 2)

Premisses:

point *A* is **outside** circle α
point *A* is **outside** circle β
point *B* is **inside** circle α
circle α is **inside** circle β

Proposed Conclusions:

point *B* is **inside** circle β
point *B* is **on** circle β

point *B* is **outside** circle β
None of the above is necessarily the case in this situation

Correct Answer:

point *B* is **inside** circle β
